# Age‐dependent changes in the gut microbiota and serum metabolome correlate with renal function and human aging

**DOI:** 10.1111/acel.14028

**Published:** 2023-11-27

**Authors:** Liang Sun, Zhiming Li, Caiyou Hu, Jiahong Ding, Qi Zhou, Guofang Pang, Zhu Wu, Ruiyue Yang, Shenghui Li, Jian Li, Jianping Cai, Yuzhe Sun, Rui Li, Hefu Zhen, Shuqin Sun, Jianmin Zhang, Mingyan Fang, Zhihua Chen, Yuan Lv, Qizhi Cao, Yanan Sun, Ranhui Gong, Zezhi Huang, Yong Duan, Hengshuo Liu, Jun Dong, Junchun Li, Jie Ruan, Haorong Lu, Benjin He, Ninghu Li, Tao Li, Wenbin Xue, Yan Li, Juan Shen, Fan Yang, Cheng Zhao, Qinghua Liang, Mingrong Zhang, Chen Chen, Huan Gong, Yong Hou, Jian Wang, Ying Zhang, Huanming Yang, Shida Zhu, Liang Xiao, Zhen Jin, Haiyun Guo, Peng Zhao, Susanne Brix, Xun Xu, Huijue Jia, Karsten Kristiansen, Ze Yang, Chao Nie

**Affiliations:** ^1^ The NHC Key Laboratory of Geriatrics Institute of Geriatric Medicine, Chinese Academy of Medical Sciences, Beijing Hospital/National Center of Gerontology of National Health Commission Beijing China; ^2^ BGI Research Shenzhen China; ^3^ China National GeneBank, BGI Research Shenzhen China; ^4^ Shenzhen Key Laboratory of Neurogenomics, BGI Research Shenzhen China; ^5^ State Key Laboratory of Genetic Engineering Collaborative Innovation Center for Genetics and Development, and Human Phenome Institute, Fudan University Shanghai China; ^6^ Jiangbin Hospital Nanning China; ^7^ Key Laboratory of Precision Nutrition and Food Quality, Department of Nutrition and Health China Agricultural University Beijing China; ^8^ School of Gerontology Binzhou Medical University Yantai China; ^9^ Office of Longevity Cultural, People's Government of Yongfu County Guilin China; ^10^ Yunnan Key Laboratory of Laboratory Medicine Kunming China; ^11^ Yunnan Institute of Experimental Diagnosis Kunming China; ^12^ Shenzhen Engineering Laboratory for Innovative Molecular Diagnostics, BGI Research Shenzhen China; ^13^ Shenzhen Engineering Laboratory of Detection and Intervention of Human Intestinal Microbiome, BGI Research Shenzhen China; ^14^ Department of Biotechnology and Biomedicine Technical University of Denmark Lyngby Denmark; ^15^ Guangdong Provincial Key Laboratory of Genome Read and Write, BGI Research Shenzhen China; ^16^ Laboratory of Genomics and Molecular Biomedicine, Department of Biology University of Copenhagen Copenhagen Denmark; ^17^ Qingdao‐Europe Advanced Institute for Life Sciences Qingdao Shandong China

**Keywords:** aging, gut microbiota, renal function, serum metabolome

## Abstract

Human aging is invariably accompanied by a decline in renal function, a process potentially exacerbated by uremic toxins originating from gut microbes. Based on a registered household Chinese Guangxi longevity cohort (*n* = 151), we conducted comprehensive profiling of the gut microbiota and serum metabolome of individuals from 22 to 111 years of age and validated the findings in two independent East Asian aging cohorts (Japan aging cohort *n* = 330, Yunnan aging cohort *n* = 80), identifying unique age‐dependent differences in the microbiota and serum metabolome. We discovered that the influence of the gut microbiota on serum metabolites intensifies with advancing age. Furthermore, mediation analyses unveiled putative causal relationships between the gut microbiota (*Escherichia coli*, *Odoribacter splanchnicus*, and *Desulfovibrio piger*) and serum metabolite markers related to impaired renal function (p‐cresol, N‐phenylacetylglutamine, 2‐oxindole, and 4‐aminohippuric acid) and aging. The fecal microbiota transplantation experiment demonstrated that the feces of elderly individuals could influence markers related to impaired renal function in the serum. Our findings reveal novel links between age‐dependent alterations in the gut microbiota and serum metabolite markers of impaired renal function, providing novel insights into the effects of microbiota‐metabolite interplay on renal function and healthy aging.

## Graphical abstract

A comprehensive analysis of the gut metagenome and serum metabolome of individuals from a Chinese longevity cohort. We discovered distinct age‐dependent differences in both microbiota and serum metabolome. Through mediation analyses and fecal microbiota transplantation, we uncovered a potential causal relationship between the microbiota and metabolite markers associated with impaired renal function and aging. These findings offer valuable insights into the impact of the microbiota‐metabolite interplay on renal function and healthy aging.

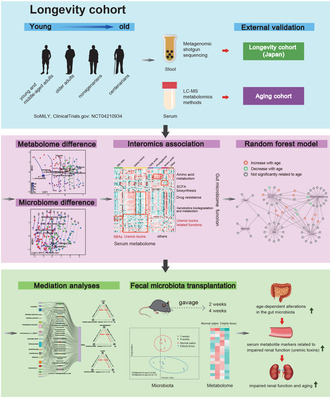

**Abbreviations**
AAAs: aromatic amino acids
BCAAs: branched‐chain amino acids
CAGs: co‐abundance gene groups
CIA: co‐inertia analysis
CKD: chronic renal disease
CKD‐EPI: chronic kidney disease epidemiology collaboration
CREA: creatinine
dbRDA: dissimilarity‐based redundancy analysis
E/B enterotype: E. coli/Bacteroides enterotype
eGFR: estimated glomerular filtration rate
FDR: false discovery rate
FMT: fecal microbiota transplantation
HCY: homocysteine
HoS: homogeneous selection
hsCRP: high‐sensitivity C‐reactive protein
iCAMP: infer community assembly mechanisms by phylogenetic bin‐based null model
KEGG: Kyoto Encyclopedia of Genes and Genomes
KO: KEGG Ortholog
LC‐MS/MS: liquid chromatography‐tandem mass spectrometry
MGSs: generation of metagenomic species
NCBI‐NT: National Center for Biotechnology Information database
PCA: principal component analysis
PE: paired‐end
PERMANOVA: permutational multivariate analysis of variance
PhILR: phylogenetic isometric log‐ratio transform
SBA: serum bile acids
SBP: systolic blood pressure
UA: uric acid
WMS: whole metagenome sequencing

## 1 INTRODUCTION

Long‐living individuals, particularly centenarians, exemplify the concept of healthy aging (Franceschi & Bonafe, 2003; Garagnani et al., 2013) and thus provide an invaluable resource for identifying novel relationships between the host and the gut microbiota in relation to aging (Marcos‐Perez et al., 2021; Santos‐Lozano et al., 2020). The global increase in the number of elderly people has spurred extensive social and healthcare concerns, posing emerging clinical challenges in relation to chronic conditions such as diabetes mellitus, renal diseases, neurological disorders, cardiovascular diseases, and neoplasms in an aging population (Chang et al., 2019; Liguori et al., 2018; O'Sullivan et al., 2017). A more profound understanding of the aging processes and the mechanisms underpinning age‐associated diseases could lay the groundwork for developing more effective healthcare strategies for elderly individuals.

With aging comes a heightened risk of renal diseases, prompting intensified research on the adaptations of renal function throughout normal aging (Denic et al., 2016; Glassock et al., 2020). These studies often involve an analysis of serum metabolites that accumulate as renal function deteriorates, potentially serving as novel biomarkers for age‐related changes in renal function (Rhee, 2018). Among these metabolites, uremic toxins have drawn significant interest, as their accumulation might signal end‐stage renal disease. Uremic toxins are biologically active compounds retained in the bodies of patients with renal failure. In healthy individuals with normal renal function, these metabolites, including indoxyl sulfate, hippuric acid, and P‐cresol, are normally excreted in the urine (Vanholder et al., 2003). However, beyond their role in renal diseases, uremic toxins have been somewhat overlooked. Given the progressive decline in renal function in older individuals, patterns of uremic toxins could act as crucial biomarkers, establishing a link between renal function and healthy aging (Kooman et al., 2014).

Recent kidney‐focused metabolomics studies have underscored the influence of diet and the gut microbiota in shaping the serum metabolome, given that many uremic metabolites require bacterial metabolism for their synthesis (Rhee, 2018; Wang et al., 2020). Specifically, uremic toxins are reportedly derived from the gut microbiota through the breakdown of diet‐derived aromatic amino acids (AAAs) and polyphenols (Ramezani et al., 2016; Wikoff et al., 2009). Supporting evidence from chronic kidney diseases and animal models has further demonstrated the critical role of the gut microbiota in renal function and the production of uremic toxins (Aronov et al., 2011; Mishima et al., 2017; Wang et al., 2020). Thus, individuals with renal failure often exhibit a severely distorted gut microbiota, leading to the rapid biosynthesis of toxic compounds, subsequently resulting in higher plasma concentrations of uremic toxins and aggravated renal disease (Wang et al., 2020).

Several cross‐sectional studies have identified gut microbiota changes that occur with aging (Biagi et al., 2016; Pang et al., 2023; Wilmanski et al., 2021; Wu et al., 2019; Zhang et al., 2021). Studies using 16S rRNA gene amplicon sequencing have indicated an association between diet‐driven microbiota alterations and health decline in aging individuals (Claesson et al., 2012) and highlighted the presence of a core microbiota of prevalent, symbiotic bacterial taxa dominated by the families *Ruminococcaceae*, *Lachnospiraceae*, and *Bacteroidaceae*, with a progressive reduction in the abundance of these core taxa with age (Biagi et al., 2016). In recent years, deep shotgun sequencing studies have reported a trend toward an increase in the abundances of *Escherichia* and *Streptococcus* with age, while the abundances of *Faecalibacterium* and *Ruminococcus* were reported to exhibit a decreasing trend (Rampelli et al., 2020; Wu et al., 2019). Notably, compared to that in other age groups, the gut microbiota of healthy centenarians is enriched with bacteria with a potential for degradation of xenobiotics (Rampelli et al., 2020) and biosynthesis of short‐chain fatty acids (Wu et al., 2019). However, whether specific interactions between the serum metabolome and gut microbiota are related to an age‐dependent decline in renal function remains largely unexplored.

The present study is based on a Chinese longevity cohort with a notable number of long‐living individuals (nonagenarians and centenarians) (Study of Microbiota in Longevity Yongfu County, SoMiLY, ClinicalTrials.gov: NCT04210934) (Sun et al., 2013). The design enabled extensive between‐group analyses of serum metabolome and gut microbiota patterns across a wide range of age groups and lifespans, identifying general age‐related changes and correlations among the gut microbiota, uremic metabolites, and renal function.

## 2 METHODS

### 2.1 Ethical approval and consent to participate

The study was approved by the Ethics Committee of Beijing Hospital and the BGI Review Board of Bioethics and Biosafety (BGI‐IRB17062). All applicable institutional regulations concerning the ethical use of information and samples from human volunteers were followed during this study. Each individual provided written informed consent. This study was registered at ClinicalTrials.gov (NCT04210934).

### 2.2 Study population

Yongfu County, located in Guangxi Province in southern China, was in the first batch of longevity towns in China, officially approved by the Chinese Gerontology Society in 2007 (Sun et al., 2013). According to site visits and government records, compared to those in other areas with more developed economies, the elderly residents in Yongfu mostly live with less dependence on modern Western medicine and health care. All the included participants were local residents and had similar social and economic backgrounds, with an average annual disposable income of approximately 10,000–20,000 yuan per person in 2016. The rural environment provided a unique research resource and potentially reduced exposure to medication, which could have a potential impact on the microbiota.

Based on an observational longevity cohort in ClinicalTrials platform (Study of Microbiota in Longevity Yongfu County, SoMiLY, NCT04210934), we recruited 151 healthy individuals from Yongfu County using nonprobability sampling and a household survey. The participants were categorized into four age groups: 29 centenarians (100–111 years old), 46 nonagenarians (90–100 years old), 41 elderly individuals (60–90 years old), and 35 young‐to‐middle‐aged adults (20–60 years old). The age information was strictly verified using China's national identity card number, double‐checked by evaluation of each generation of children, and further validated by asking participants to recall their life events in home visits. The exclusion criteria included self‐reported antibiotic use within 1 month, hospitalization for any reason in the last 3 months, acute major diseases or disabilities, typical dementia‐related inability to communicate, and intake of any drug potentially affecting the microbiota, especially oral antidiabetic drugs, lipid‐lowering agents, and cancer chemotherapeutic agents within 3 months.

In addition, two independent external cohorts with East Asian origins, the Yunnan aging cohort (*n* = 80) and the Japan aging cohort (*n* = 330), were used to validate serum metabolomic and fecal metagenomic features identified in the Guangxi aging cohort (Table S1). Yunnan and Guangxi are contiguous and both located in South China, and the residents exhibit more similar geographic features, dietary habits, and living habits than Caucasians and Africans. Eighty participants undergoing health management were included; the routine health indicators of these participants were within the reference range, and they exhibited an overall good health status. Their ages spanned four stages, termed young and middle‐aged (20–45 years old), young‐old (60–69 years old), middle‐old (70–79 years old), and old–old (80–89 years old), with 20 individuals in each stage (equally divided between males and females). In addition, the Japan aging datasets were retrieved via the public database (PRJNA675598) (Sato et al., 2021), comprising 330 gut microbial metagenomes from Japanese adults who had overall similar gender and age distributions as the Guangxi longevity cohort.

### 2.3 Blood and fecal sample collection

Blood and fecal samples of participants were collected at home during a household survey and transferred to the clinical laboratory in the Yongfu People's Hospital on dry ice. Serum was isolated after centrifugation twice (3000 rpm, 10 min and 12,000 rpm, 5 min), and stored at −80°C until used for metabolomics analysis. Fresh fecal samples were obtained at home at the same time as blood collection. After excretion, all pretreatment was performed within 10 min to ensure that the feces were relatively fresh. The whole operation process was carried out while keeping the samples on dry ice to ensure a low‐temperature environment. We used fecal collection kits (MGIEasy) to collect fresh fecal samples and stored them in an ice box with dry ice; the samples were subsequently stored at −80°C until DNA extraction and metagenomics analysis. To control for possible contaminants during handling and nucleic acid preparation, we included a blank control every day for each batch, wherein the blank swab was likewise immersed in the fecal preservation solution.

### 2.4 General information and clinical phenotypes

The general parameters recorded included age, sex, weight, height, smoking status, drinking habits, systolic blood pressure (SBP), and diastolic blood pressure (see Table S1 for details). Six clinical phenotypes were evaluated, including glycometabolism (hemoglobin, C‐peptide), lipid metabolism (triglycerides, total cholesterol, high‐density lipoprotein cholesterol, and low‐density lipoprotein cholesterol), inflammation (high‐sensitivity C‐reactive protein [hsCRP]), redox state (superoxide dismutase), cardiovascular biomarkers (beta‐hydroxybutyrate, homocysteine) and renal function [creatinine [CREA], UREA, uric acid [UA]). For calculation of the estimated glomerular filtration rate (eGFR) (mL/min per 1.73 m^2^), the Chronic Kidney Disease Epidemiology Collaboration (CKD‐EPI) (Matsushita et al., 2012) equations were used as follows:

$$\text{Female with}\;\mathrm{SCr}\leq 0.7:\text{eGFR}=144\times {\left(0.993\right)}^{\mathrm{age}}\times {\left(\mathrm{SCr}/0.7\right)}^{-0.329}$$

$$\text{Female with}\;\mathrm{SCr}>0.7:\text{eGFR}=144\times {\left(0.993\right)}^{\mathrm{age}}\times {\left(\mathrm{SCr}/0.7\right)}^{-1.209}$$

$$\text{Male with}\;\mathrm{SCr}\leq 0.9:\text{eGFR}=141\times {\left(0.993\right)}^{\mathrm{age}}\times {\left(\mathrm{SCr}/0.9\right)}^{-0.4111}$$

$$\text{Male with}\;\mathrm{SCr}>0.9:\text{eGFR}=141\times {\left(0.993\right)}^{\mathrm{age}}\times {\left(\mathrm{SCr}/0.9\right)}^{-1.209}$$

### 2.5 Leukocyte telomere length measurement

Peripheral blood leucocyte DNA extraction was performed using a Whole Blood DNA Extraction kit (BioTeke, Beijing, China) and evaluated using a Qubit fluorometer. Telomere length was determined as the relative ratio of the telomere repeat copy number to a single copy gene copy number (T/S ratio) using the quantitative PCR (Cawthon, 2002). Each sample was tested for telomeres (T) and the single copy gene (S), RNaseP, with 3 replicates for each. The self‐configured gDNA mixed samples were used as standards to eliminate the systematic errors of the results of different batches. The formula for calculating the T/S value was: T/S = 2^−∆∆Ct^; ∆∆Ct = ∆Ctsample − ∆Ctstandard; ∆Ct = Ct(T) − Ct(S).

### 2.6 Metabolite profiling of human serum samples

#### 2.6.1 Internal standards (1)

D3‐L‐methionine (100 ppm, TRC, Canada), 13C9‐phenylalanine (100 ppm, CIL, USA), D6‐L‐2‐aminobutyric acid (100 ppm, TRC, Canada), D4‐L‐alanine (100 ppm, TRC, Canada), 13C4‐L‐threonine (100 ppm, CIL, USA), D3‐L‐aspartic acid (100 ppm, TRC, Canada), and 13C6‐L‐arginine (100 ppm, CIL, USA).

#### 2.6.2 SPLASH internal standards (2)

The stock concentrations of each lipid standard were as follows: LPC 18:1(d7), 25 μg/mL; LPE 18:1(d7), 5 μg/mL; PC 15:0–18:1(d7), 160 μg/mL; PE 15:0–18:1(d7), 5 μg/mL; PG 15:0 18:1(d7), 30 μg/mL; PS 15:0–18:1(d7), 5 μg/mL; PI 15:0–18:1(d7), 10 μg/mL; PA 15:0–18:1(d7), 7 μg/mL; SM d18:1–18:1(d9), 30 μg/mL; cholesterol(d7), 100 μg/mL; CE 18:1(d7), 350 μg/mL; MG 18:1(d7), 2 μg/mL; DG 15:0–18:1(d7), 10 μg/mL; and TG 15:0–18:1(d7)–15:0, 55 μg/mL. Methanol (A454‐4), acetonitrile (A996‐4), and the above substances were of LC–MS grade. Formic acid ammonium salt (17843‐250G; Honeywell Fluka, USA), and formic acid (50144‐5 g0mL DIMKA, USA) were used, and water was supplied by a water purification system.

#### 2.6.3 Metabolite extraction

After thawing a sample slowly at 4°C, 100 μL was placed into a well of a 96‐well plate, and 300 μL of extraction solvent (2:1 methanol:ACN (V/V) precooled at −20°C), 10 μL of internal standard 1 and 10 μL of internal standard 2 were added. The mixture was vortexed for 1 min, incubated at −20°C for 2 h, and then centrifuged at 4000 rpm for 20 min at 4°C. After centrifugation, 300 μL of the supernatant was subjected to freeze drying, and the residue was resuspended in 150 μL of 1:1 methanol:H_2_O (V/V). The mixture was vortexed for 1 min and centrifuged at 4000 rpm for 30 min at 4°C, and the supernatant was placed into a sample bottle. Ten microliters of the supernatant from each sample was mixed as the QC sample to evaluate the repeatability and stability of LC–MS analysis.

#### 2.6.4 Liquid chromatography–tandem mass spectrometry (LC–MS/MS)‐chromatographic conditions

We used a Waters 2D UPLC (Waters USA) tandem Q Exactive high‐resolution mass spectrometer (Thermo Fisher Scientific, USA) to separate and detect metabolites.

#### 2.6.5 Chromatographic conditions

A BEH C18 column (1.7 μm 2.1 × 100 mm, Waters, USA) was used. In positive ion mode, the mobile phase was a water solution containing 0.1% formic acid (A) and 100% methanol containing 0.1% formic acid (B). In negative ion mode, the mobile phase was an aqueous solution containing 10 mM ammonia formate (A) and 95% methanol containing 10 mM ammonia formate (B). The following gradient was used for elution in both ionization modes: 0–1 min, 2% B; 1–9 min, 2%–98% B; 9–12 min, 98% B; 12–12.1 min, 98% B; 12.1–15 min, 2% B. The flow rate was 0.35 mL/min, the column temperature was 45°C, and the injection volume was 5 μL.

#### 2.6.6 Mass spectrometric conditions

Primary and secondary mass spectrometric data were collected by a Q Exactive mass spectrometer (Thermo Fisher Scientific, USA). The mass‐to‐charge ratio scan range was m/z 70–1050, the first‐order resolution was 70,000, the AGC was 3e6, and the maximum injection time was 50 ms. According to the precursor ion signal strength, the top 3 ions were selected for fragmentation, and secondary mass spectral data were acquired. The MS/MS resolution was 17,500, AGC was 1e5, maximum injection time was 50 ms, and stepped collision energies were 20, 40, and 60 eV. The parameters of the electrospray ionization source were as follows: sheath gas flow rate, 40 L/h; aux gas flow rate, 10 L/h; spray voltage (|kV|), 3.80 in positive ionization mode and 3.20 in positive ionization mode; capillary temperature, 320°C; and aux gas heater temperature, 350°C.

#### 2.6.7 Identification of metabolites and data analysis

Compound Discoverer 3.0 (Thermo Fisher Scientific, USA) software was used to process LC–MS/MS data, including for peak extraction, peak alignment, and compound identification. After data processing, a total of 35,652 metabolites were detected. For further LC–MS/MS analysis, known metabolite annotation was performed according to an in‐house HMDB and the Kyoto Encyclopedia of Genes and Genomes (KEGG) database. A total of 365 out of the 35,652 metabolites were structurally identified and annotated according to the in‐house LC–MS/MS database.

### 2.7 Fecal DNA extraction and metagenomic sequencing

Total DNA was extracted from approximately 400–500 mg of feces using the MetaHIT protocol (Qin et al., 2012). The DNA concentration was estimated by a Qubit instrument (Invitrogen). DNA library construction and sequencing using the BGISEQ‐500 platform were performed as described previously (Fang et al., 2018; Li et al., 2021). Five hundred nanograms of input DNA was used for library formation and fragmented ultrasonically with a Covaris E220 (Covaris, Brighton, UK), yielding 300 to 700 bp fragments. We constructed one paired‐end (PE) library for each sample, followed by high‐throughput sequencing with PE reads of 2 × 100 bp. We used the documented workflow (Fang et al., 2018; Li et al., 2021) for sequencing data quality control. Human genomic DNA reads were identified using bwa‐mem2 (Vasimuddin et al., 2019), and reads were removed if they shared >95% sequence identity with the human genome reference sequence (hg38).

During the sampling process, we designed a blank control every day and placed the blank swab into the fecal preservation solution. No DNA was found in the blank control during DNA extraction.

### 2.8 Gene catalog construction and gene annotation

#### 2.8.1 Gene catalog construction

Based on the whole metagenome sequencing (WMS) data of all individual fecal samples, a de novo gene catalog was constructed (Liyanage et al., 2015). High‐quality reads of each sample were used for de novo assembly with Megahit (version 1.1.2) (Li et al., 2015), which generated the initial assembly results based on different k‐mer sizes (*k* = 21, 41, 61, 81) and then merged them. Ab initio gene identification of assembled contigs was conducted using MetaGeneMark (Zhu et al., 2010). Then, cd‐hit (version 4.5.4) (Fu et al., 2012) clustered the predicted genes at the nucleotide level, and genes with more than 90% overlap and more than 95% homology were treated as redundant (Human Microbiome Project Consortium, 2012). Finally, we obtained a nonredundant gene catalog of 4,140,158 genes, of which 1,154,273 were partial ORFs (27.9%).

#### 2.8.2 Quantification of genes

The high‐quality sequences were mapped to the above nonredundant gene catalog using bwa‐mem2 (Vasimuddin et al., 2019) with the criterion of identity >90%. Based on the alignment result, the relative abundance of gut microbial genes was evaluated by the same method as that used in previous microbiome studies (Li et al., 2021; Qin et al., 2012).

#### 2.8.3 Taxonomic classification of genes

The nonredundant gene catalog was compared with sequences in the National Center for Biotechnology Information database (NCBI‐NT, downloaded at Aug. 2018) using BLASTN (v2.7.1) by the parameter “word_size 16‐evalue 1e − 10”. Alignments were filtered to require at least 70% query coverage. If one gene matched two or more different NCBI‐NT sequences with exactly the same bit‐score but from different species, we performed statistics on multiple best‐hits (from the NCBI‐NT database) mapping for the same gene, including the number of species present, the number of occurrences of each species, and the average similarity of the same species. After completion of the statistical analysis, the species annotation with the highest frequency and the highest average similarity was used as the annotation for the gene (shown in the table below). When a gene was annotated to different species based on the NCBI‐NT database, the highest identity of the species annotated for that gene in the BLASTN results was prioritized. Ninety‐five percent identity was used as the critical value for species assignment, 85% identity was used as the critical value for genus assignment, and 65% identity was used as the critical value for phylum allocation (Arumugam et al., 2011). A total of 1.97 million genes were classified and annotated taxonomically.

#### 2.8.4 Functional annotation of genes

We used BLASTP (v2.7.1) with the parameter “word_size 16‐evalue 1e − 6” to align the putative amino acid sequence translated from the gene catalog with the protein or domain in the KEGG database (version 84.0, excluding animal or plant genes), and alignments were filtered to require at least 30% alignment identity and 70% query coverage. Each putative amino acid sequence was assigned a KEGG ortholog based on the best‐hit gene in the KEGG Ortholog (KO) database. Using the above method, 3,101,635 genes in the combined gene catalog were assigned to the KEGG database.

#### 2.8.5 Construction of gene and KO profiles

For gene and KO profiling, we used a previously reported method (Li et al., 2021). In brief, the relative abundance of a KO was calculated as the total sum of the relative abundance of its cognate genes.

### 2.9 Generation of metagenomic species (MGSs) and taxonomic classification

The generation of MGS was performed as previously described (Nielsen et al., 2014). Co‐abundance gene groups (CAGs) were established using “cc.bin” (Nielsen et al., 2014) (default parameters) from the correlation clusters of sample abundance profiles by a canopy‐based algorithm. Canopy‐based clustering of the gene catalog was performed by iteratively picking a seed gene among the not yet clustered genes and aggregating genes with abundance profiles within a fixed distance from the seed gene abundance profile (Pearson correlation coefficient >0.9 and Spearman's rank correlation coefficient >0.6) into the seed canopy. Canopies with median abundance profiles within a distance of 0.97 Pearson correlation coefficient from one another were merged. Canopies with 2 or fewer genes, for which the canopy abundance signal from any three samples constituted 90% or more of the total signal across all samples, for which the median profile was detected in less than four samples, or for which one sample made up 90% of the total signal were discarded for having insufficient supporting evidence. Canopies that passed these criteria were called CAGs. CAGs with more than 700 genes are also referred to as MGSs.

Taxonomic classification was based on the NCBI‐NT database as previously described (Glassock et al., 2017). Assignment to a species required that 90% of the genes in an MGS aligned with this species' genome with 95% identity and 70% overlap in the query. Assigning an MGS to a genus required 80% of its genes to align with a genome with 85% identity in both DNA and protein sequences. MGSs that did not meet this condition were unclassified.

We calculated the MGS profile using the abundance of each gene in the original gene catalog. The abundance of MGSs was determined by taking the median of the relative abundances of all genes within a cluster. Additionally, we conducted a taxonomic and compositional analysis of the metagenome by the Metaphlan4 (V 4.0.6) (Blanco‐Míguez et al., 2023) tool using the MetaPhlAn database of marker genes mpa_vOct22_CHOCOPhlAnSGB_202212.

### 2.10 Alpha diversity and enterotype analysis

For each sample, we calculated the Shannon (2001) entropy index, and the median Shannon entropy was used for comparisons between samples:

$$H^{\prime }=-\sum_{i=1}^{S}a_{i}{\mathrm{lna}}_{i},$$

where *S* is the number of genes and *a*_*i*_ is the relative abundance of gene *i*. A high α‐diversity indicates a high evenness or diversity of genes present in the sample. Enterotyping was performed based on a previously described method (Arumugam et al., 2011) (https://enterotype.embl.de/enterotypes.html).

### 2.11 Infer community assembly mechanisms by phylogenetic bin‐based null model (iCAMP) analysis

iCAMP was used to investigate the assembly mechanisms of different microorganism groups (Ning et al., 2020). The R code for iCAMP was available as an open‐source R package, “iCAMP,” which can be downloaded from the Comprehensive R Archive Network (CRAN, https://cran.r‐project.org/). By using iCAMP, five assembly mechanisms of different microorganism groups were identified, including homogeneous selection (HoS), heterogeneous selection, dispersal limitation (DL), homogenizing dispersal, and drift (DR).

### 2.12 Phylogenetic isometric log‐ratio transform (PhILR) analysis

PhILR (Silverman et al., 2017) analysis incorporates microbial evolutionary models with the isometric log‐ratio transform to safely allow off‐the‐shelf statistical tools to be applied to microbiota surveys. The R code for PhILR was available as an open‐source R package, “philr,” which can be downloaded from “https://bioconductor.org/packages/devel/bioc/vignettes/philr/inst/doc/philr‐intro.html#ordination‐in‐philr‐space”. This analysis combines the abundance table of species composition and phylogenetic relationships among species. We further conducted principal component analysis (PCA) using PhILR‐converted data.

### 2.13 Bidirectional mediation analysis

For microbial features associated with metabolites and aging, we first checked whether the microbial features were associated with the metabolite using Spearman correlation (*p* < 0.05). Next, we carried out bidirectional mediation analysis with interactions (*y* = *x* + *m* + *x* × *m*, where *y* is the outcome, *x* is the variable and *m* represents the mediator) between mediator and outcome using the mediate function from mediation (version 4.5.0) to infer the mediation effect of serum metabolites and the gut microbiota on aging (Tingley et al., 2014).

### 2.14 Random forest models

Age and health status were predicted (random forest 4.6‐12 package Breiman, 2001) using the MGS profiles.

#### 2.14.1 Variable selection

First, the random forest regression model was used to predict the metabolites, and the largest variable of IncMSE was selected as the first variable. Second, the first variable and the remaining variables were combined into two variables to predict the metabolites, and the *Q* ^2^ values of the predicted results were calculated and compared (the largest was the second variable of choice). Further variables were added iteratively in the same way until *Q* ^2^ no longer increased.

$$Q^{2}=1-\frac{\sum {\left(y_{i}-\hat{y_{i}}\right)}^{2}}{\sum {\left(y_{i}-\bar{y_{i}}\right)}^{2}}.$$

Here, *y*_i_ and $\hat{y_{i}}$ are the i‐th observation value and the predicted value of the fecal metabolites, respectively.

#### 2.14.2 Module training

The metabolites were predicted by the variables selected above, *Q* ^2^ was calculated, and the variables of importance were obtained.

#### 2.14.3 Cross‐validation

We used the leave‐one‐out cross‐validation method. Each sample was separately used as a validation set, and the remaining 150 samples were used as a training set. *Q* ^2^ was calculated by the predicted value and the experimental value. Cross‐validation was used in all random forest modeling processes.

### 2.15 The fecal microbiota transplantation (FMT)

Male C57BL/6 J mice (8 weeks), purchased from SPF biotechnology company. (Beijing, China) were provided with food and water ad libitum. Environmental temperature was kept at 23 ± 1°C with a 12‐h light/dark cycle. Before transplantation, mice were treated for three consecutive days with 200 μL of an antibiotic cocktail (with each daily dose being administered by oral gavage after a 6 h fast) that contained 1 mg/mL ampicillin, 0.5 mg/mL neomycin, 0.5 g/L vancomycin and 1 g/L metronidazole according to the preciously published protocol (Bárcena et al., 2019).

For FMT, mice were randomized into the following groups (*n* = 10 per group): control (gavage saline), FMT‐aged (feces from aged donors). Fecal material was collected from 20 community‐dwelling elderly individuals aged 80 and over who had not taken any antibiotics in the past 3 months. Equal amounts of fecal material from all donors were pooled and 10% (w/v) fecal suspension was prepared. Thereafter, mice were given 200 μL human fecal suspension three times a week for 4 weeks by gavage according to precious studies, starting the first day after the antibiotic cycle until sacrifice (Liu et al., 2020). At the end of weeks 2 and 4 of FMT, fresh feces were collected for microbiota analysis, and cardiac blood was collected for serum separation for metabolomics analysis.

### 2.16 Statistical analysis

#### 2.16.1 Multivariate analysis

Multivariate statistical analysis (PCA and dissimilarity‐based redundancy analysis [dbRDA]) was used to distinguish and analyze serum metabolites and gut microbiome samples from individuals of different ages. PCA was performed by using the ade4 package (Thioulouse et al., 2018) in the R platform. Based on Bray–Curtis dissimilarity, dbRDA (Legendre & Anderson, 1999) was performed by using capscale (McArdle & Anderson, 2001) (a function in the vegan package in R). We show the dbRDA plots of the main constraint axis (CAP1) and the main multidimensional scale (mds1) in the main text.

#### 2.16.2 Permutational multivariate analysis of variance (PERMANOVA) analysis

In this study, we integrated a multiomics method (Price et al., 2017) to analyze the relationship among the serum metabolome, gut microbiome and phenome. We performed PERMANOVA to determine whether the omics datasets could affect each other. To evaluate the variance proportion of the serum metabolism data interpreted from the gut microbiome and phenome, first, the adonis function of the R package vegan was used to estimate the effect size (*R* ^2^) between each variable of the gut microbiome and phenome and the serum metabolome. Only the variables with a significant impact on the serum metabolome (*p* < 0.05, 999 permutations) were considered. Then, to remove redundant variables, Pearson correlation coefficients between variables were calculated, and variables with correlation coefficients greater than 0.5 were removed. Finally, the adonis function was used to calculate the combined effect size based on all nonredundant variables.

#### 2.16.3 Co‐inertia analysis (CIA)

To assess the consistency of samples in the projection of the serum metabolome and host phenome, we performed CIA (the parameter was “scannf = FALSE, nf = 2”) (Wang et al., 2020) on serum metabolites and phenome profiles of all samples. The CIA plots in the main text (Figure S5a) were generated by R software (vegan package, *CIA* function).

#### 2.16.4 Age correction

The association between any two groups may be affected by age, so we used age as a covariate to correct the influence of age on the two groups. We used the pcor.test (Kim, 2015) function in the R software ppcor package to correct for the influence of age.

#### 2.16.5 Hypothesis testing and multiple test correction

Wilcoxon rank‐sum tests (Bauer, 1972) were conducted to detect differences in the gut microbial and serum metabolome characteristics, including the Shannon index, host phenotype, and serum metabolites. Kruskal–Wallis tests (Hollander et al., 2013) were performed to assess the differences in gut microbial composition, function, serum metabolites, and continuous phenotypic variables among different age groups. False discovery rate (FDR) adjustment was performed by the Benjamin‐Hochberg method (Benjamini & Hochberg, 1995) (using the R package *p.adjust*), and the local FDR is provided in the article.

#### 2.16.6 Power analysis

We evaluated the power of each correlation analysis. We used the pwr.r.test function in the R software pwr package to analyze the power (the parameter was alpha = 0.05, alternative = “two.sided”). The power of the Guangxi longevity cohort was more than 80% for each of the combined parameters (Table S15).

## 3 RESULTS

### 3.1 The cohorts

We collected and analyzed stool and serum samples from individuals living in Yongfu County, Guangxi, China, known for a high proportion of nonagenarians and centenarians (Sun et al., 2015). The standard clinical parameters were collected, and are detailed in Table S1. We assessed the relative telomere length in 108 individuals and found an inverse correlation with age (Spearman's *r* = −0.2, *p* = 0.02) (Figure S1, Table S2), confirming prior findings about age‐dependent telomere shortening (Aviv et al., 2001). In addition, we determined that a high proportion of the elderly individuals had increased hsCRP levels (Spearman's *r* = 0.312, *p* < 0.001) (Figure S1), consistent with previously published associations between these parameters and age (Glassock et al., 2017; Goto et al., 2012; Kooman et al., 2014).

Each stool sample was subjected to shotgun metagenomic sequencing followed by profiling of the microbial community composition and inferred functional potential. To assess whether the age‐related gut microbial characteristics in the Guangxi longevity cohort could be generalized to other populations, a total of 330 adult gut microbial metagenomes from Japanese adults, who had overall similar gender and age distributions as the Guangxi longevity cohort, were retrieved via the public database (PRJNA675598) (Sato et al., 2021) (Table S1).

We used LC–MS/MS to analyze metabolites in 136 out of the 151 serum samples. The same protocol was used to analyze serum samples from an additional 80 individuals (aged 20 to 80 years) from the “Yunnan aging cohort” in southern China. LC–MS/MS metabolite profiling was conducted in a nontargeted mode using sensitive high‐resolution mass spectrometry that captured known and uncharacterized metabolites, including metabolites potentially produced by gut bacterial species. We only included the known identified metabolites in the analysis in this study.

### 3.2 Composition and functional potential of the gut microbiota across different age groups

We obtained an average of 12.6 Gb of sequencing data per fecal sample (Table S3). The sequencing data were utilized to assemble a gene catalog of 4.14 million nonredundant microbial genes, representing the microbiome of our Guangxi longevity cohort with an average of 70.16% mapping reads in each sample (Table S3). The genes were further annotated into 6631 KEGG functional categories and classified into 601 bacterial species identified as MGSs, of which only approximately half could be ascribed to known genera, suggesting the presence of a considerable number of novel genera in the dataset (Table S4).

**FIGURE 1:**
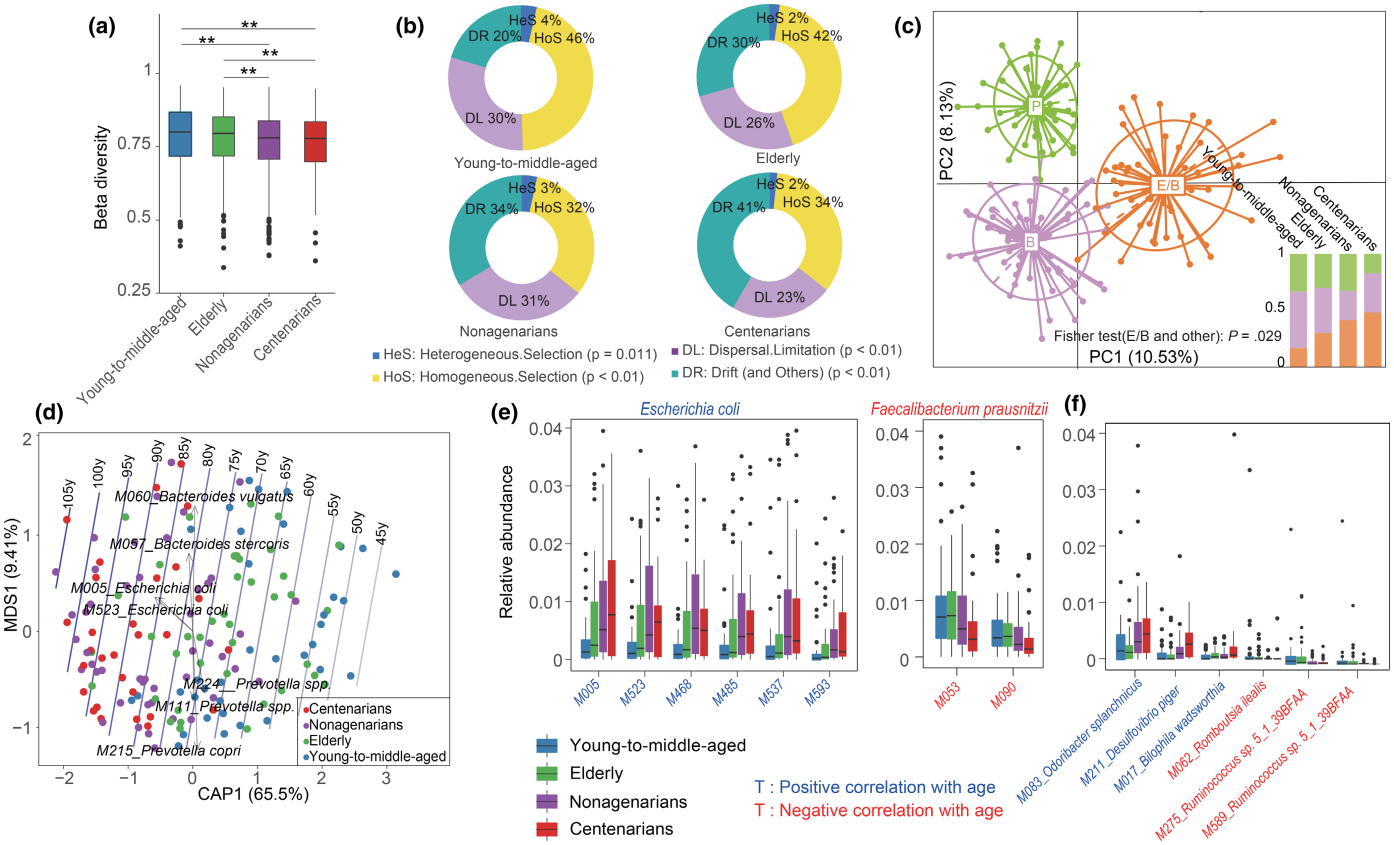
Comparison of the gut microbiota among young‐to‐middle‐aged adults, elderly individuals, nonagenarians, and centenarians. (a) Box plots of intragroup beta diversity based on MGSs in young‐to‐middle‐aged adults, elderly individuals, nonagenarians, and centenarians (*p < 0.05, **p < 0.01; Wilcoxon rank‐sum test). (b) Relative importance of different ecological processes in response to aging. (c) Enterotype analysis using the 151 profiled metagenomes. The degree of separation between individuals is shown using between‐class analysis and PCA (see Section 2). The histogram in the lower right corner shows the proportion of the three enterotypes in each age group. P: *Prevotella*, B: *Bacteroides*: E/B: *E. coli*/*Bacteroides*. (d) dbRDA of MGSs of young‐to‐middle‐aged adults, elderly individuals, nonagenarians and centenarians. (e–f) Box plots displaying the abundance of significantly different bacterial species among young‐to‐middle‐aged adults, elderly individuals, nonagenarians and centenarians. Bacterial names in red are negatively correlated with age, and those in blue are positively correlated with age.

Echoing findings from prior research on Sardinian centenarians (Wu et al., 2019), the diversity (evaluated by the Shannon diversity index) of gut microbial genes was similar across the four age groups (Wilcoxon rank‐sum test, *p* > 0.05; Figure S2a), indicating that gene diversity remained relatively stable regardless of ethnicity or age. Nevertheless, unlike previous studies (Claesson et al., 2012), we noted a higher β diversity in the gut microbiota of young‐to‐middle‐aged and elderly individuals, signaling a more varied community structure within these groups compared to their long‐living counterparts (nonagenarians and centenarians). This points to a convergence of gut microbiota composition in long‐living individuals (Figure 1a).

Based on iCAMP (Ning et al., 2020) analysis, HoS, DL, and drift were more important than other processes in bacterial community assembly, with average relative importance values of 32%–46%, 23%–30% and 20%–41%, respectively (Figure 1b). Aging significantly influenced the relative importance of these processes (*p* < 0.01, permutational ANOVA), with advancing age reducing the impact of HoS and increasing drift. An analysis of enterotypes (Arumugam et al., 2011) revealed that three enterotypes characterized the cohort, one driven by *Bacteroides*, one by *Prevotella*, and surprisingly, one by *E. coli* (E enterotype), with the last one contrasting previous findings. *Bacteroides* was found to be the second driving genus of the E enterotype, which we accordingly named the E/B enterotype (*E. coli/Bacteroides* enterotype) (Figure 1c, Table S5). The absence of Firmicutes‐driven enterotypes might be attributed to limited sample size or specific dietary conditions in Yongfu County, affecting this age‐diverse cohort. Notably, comparisons of young‐to‐middle‐aged adults, elderly individuals, nonagenarians, and centenarians revealed that the distribution of the E/B enterotype in longer‐living individuals was skewed toward higher occurrence in nonagenarians and centenarians (Figure 1c, Fisher's exact test, *p* < 0.05), implying that the gut microbiota may change during life, or that the gut microbiota may have settled differently in the young generations.

Both dbRDA and PhILR based on MGSs demonstrated a clear separation of dominant microbial species among the different age groups (Figure 1d, Figure S2b, PERMANOVA *p* = 0.002). A comparison of the abundances of bacterial species across these age groups revealed 22 age‐associated MGSs (Spearman's correlation, FDR *q* < 0.05, Table S6). Interestingly, 18 of these age‐associated MGSs (Table S6) were also found to be age‐associated in a recent Japanese centenarian cohort (Sato et al., 2021). The genetics and dietary habits of the general Chinese and Japanese populations are relatively similar to each other compared to those of Caucasians and Africans. Thus, combined, the results indicate that the compositional changes in identified microbes seem to converge as people gradually age regardless of ethnicity. In line with previous cross‐sectional studies, the six identified *E. coli* MGSs were significantly enriched in the oldest individuals in the Guangxi longevity cohort, whereas the two *Faecalibacterium prausnitzii* MGSs were enriched in the youngest individuals (Biagi et al., 2016; Wu et al., 2019) (Spearman's correlation, FDR *q* < 0.05; Figure 1e, Table S6). Moreover, we found that *O. splanchnicus*, *D. piger*, *Bilophila wadsworthia*, *Enterobacter cloacae*, and *Lactococcus garvieae* were enriched in the oldest individuals, while *Romboutsia ilealis* and *Ruminococcus* spp. were more abundant in the youngest individuals (Spearman's correlation, *FDR q* < 0.05; Figure 1e, Table S6). Utilizing Metaphlan4 (Blanco‐Míguez et al., 2023) for microbial composition and relative abundance evaluation, we corroborated our above findings, noting that *Anaerostipes hadrus*, *Clostridium symbiosum*, *E. coli*, and *O. splanchnicus* were closely related to aging (Spearman's correlation, FDR *q* < 0.05; Table S6).

KO analysis related to the gut microbiota also exhibited age‐related differences (Figure S2c). The potential for degradation of xenobiotics and multidrug resistance of the gut microbiota was significantly enhanced in older individuals (Figure S2d). In contrast, in the older age groups, a reduced potential for the biosynthesis of the branched‐chain amino acids (BCAAs), leucine, and isoleucine, was observed, while the potential for degradation of the BCAA valine was increased (Figure S2d). More importantly, we observed that the abundance of KOs related to tyrosine, tryptophan, and phenylalanine metabolic pathways, conferring the ability to produce markers of impaired renal functions (indole, phenol, phenylacetylglutamine, and p‐cresol), increased with age (Figure S2e). Consistent with previous reports (Sato et al., 2021), an increased potential of the gut microbiota for the biosynthesis of secondary bile acids as well acetyl‐CoA via the malonate semialdehyde pathway was associated with age (Figure S2d). Together, these findings revealed several age‐dependent differences in the functional potential of the gut microbiota.

### 3.3 Age‐related differences in the serum metabolome

**FIGURE 2:**
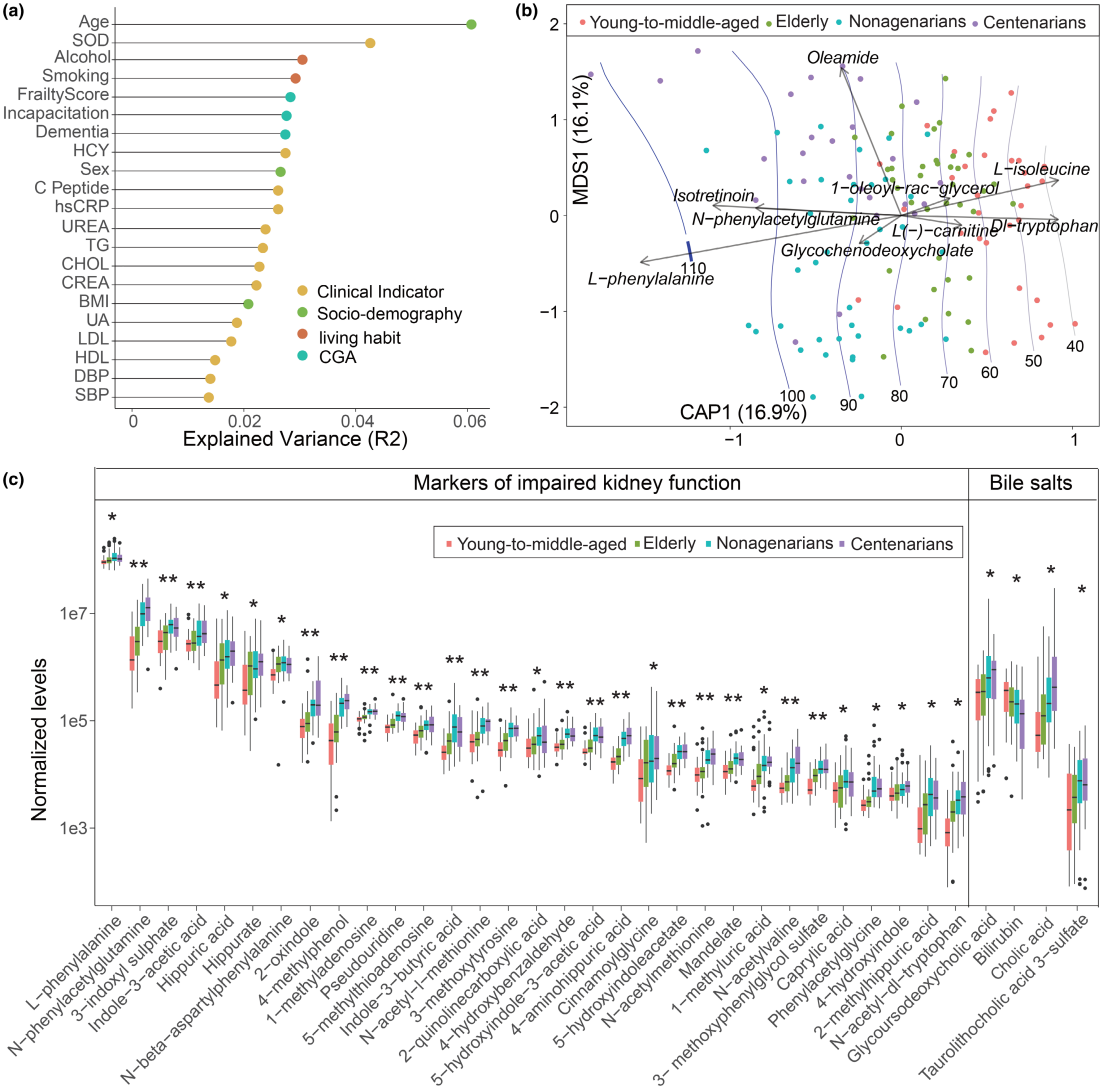
Broad changes in serum metabolomic profiles by age. (a) Effect size of phenotypic indices that significantly explain the variance (*R* ^2^) in the serum metabolome (*adonis p* < 0.05). This analysis was based on all subjects, including young‐to‐middle‐aged adults, elderly individuals, nonagenarians, and centenarians. (b) dbRDA of serum metabolites according to age. Metabolites that were identified as the main contributors to age distinction are indicated with arrows. (c) Boxplot displaying serum metabolites that differ significantly in abundance (specific uremic toxins and bile salts) among young‐to‐middle‐aged adults, elderly individuals, nonagenarians. and centenarians. CHOL, total cholesterol; CREA, creatinine; DBP, diastolic blood pressure; HCY, homocysteine; hsCRP, high‐sensitivity c‐reactive protein; HDL, high‐density lipoprotein; LDL, low‐density lipoprotein; SBP, systolic pressure; SOD, superoxide dismutase; TG, triglycerides; UA, uric acid. *p < 0.05; **p < 0.001.

We identified age as the primary factor explaining the variance (~6%) within the serum metabolome of the subjects in the Guangxi longevity cohort (Figure 2a, PERMANOVA, *p* < 0.001). Notably, lifestyle choices such as smoking and alcohol consumption also influenced the serum metabolome, albeit to a lesser extent (Figure 2a, PERMANOVA, *p* < 0.001). Moreover, we also observed distinctive serum metabolome patterns across the different age groups (Figure 2b). Specifically, 128 of 365 metabolites were significantly correlated with age (Figure 2c, Table S7, Spearman's correlation, FDR *q* < 0.05). These metabolites spanned a wide spectrum, encompassing lipids, amino acids, bile salts, prostaglandins, and other metabolites (Table S7). Of these metabolites, 91 correlated positively with age (Spearman's correlation, FDR *q* < 0.05). In particular, the levels of 31 markers related to impaired renal function (including p‐cresol, hippuric acid, N‐phenylacetylglutamine, 3‐indoxyl sulfate, and 2‐oxindole) increased progressively with age (Figure 2c, Table S7, Spearman's correlation, FDR *q* < 0.05). Markers of impaired renal function, such as 3‐indoxyl sulfate, p‐cresol, hippuric acid, and N‐phenylacetylglutamine have been reported to be produced by the gut microbiota via the degradation of diet‐derived AAAs (Mishima et al., 2015), polyphenols, and choline (Ramezani et al., 2016; Wikoff et al., 2009) (Figure S3). 3‐Indoxyl sulfate and p‐cresol have been experimentally proven to induce kidney fibrosis and cause significant kidney tubular damage in rat models of CKD (Vanholder et al., 2014). *E. coli* enriched in the Guangxi longevity cohort has also been reported to be involved in the transformation of polyphenols into benzoic acid, 4‐hydroxybenzoic acid, or hippuric acid in the human gut (Moco et al., 2012).

Furthermore, we observed variability in the level of cholic acid across all age groups (Figure 2c). Altered profiles of circulating bile acids have been reported to be associated with renal diseases (Rajani & Jia, 2018) and are known to be modified by the gut microbiota (Jie et al., 2017). The diminished level of amino acids mainly reflected a decline in tryptophan with age (Table S7), potentially related to the ability of the gut microbiota to metabolize tryptophan to produce indole and p‐cresol (Agus et al., 2018; Figure S3). Other features, such as prostaglandins, carnitine derivatives, and disease‐related metabolites, were also associated positively with age, whereas the amounts of hormones, amino acids, nicotine, and their derivatives decreased (Table S6). The diminished production of amino acids was mainly reflected as a decline in tryptophan with age (Table S6).

Of the 128 metabolites significantly associated with age in the Guangxi longevity cohort, we further examined the relationship between the relative abundance of these metabolites and age in the Yunnan aging cohort using correlation analysis. Thirty‐five of these 128 metabolites also exhibited a significant correlation with age in the Yunnan aging cohort, including N‐phenylacylglutamine and 3‐methoxytyrosine (Table S7, Spearman's correlation, FDR *q* < 0.05). A total of 102 of 128 metabolites exhibited congruent trends with age in the Guangxi longevity cohort and the Yunnan aging cohort (Figure S4, Table S7). Notably, 28 out of these 102 metabolites are markers of impaired renal function, highlighting a possible involvement of deteriorating renal function in the aging process (Table S7).

**FIGURE 3:**
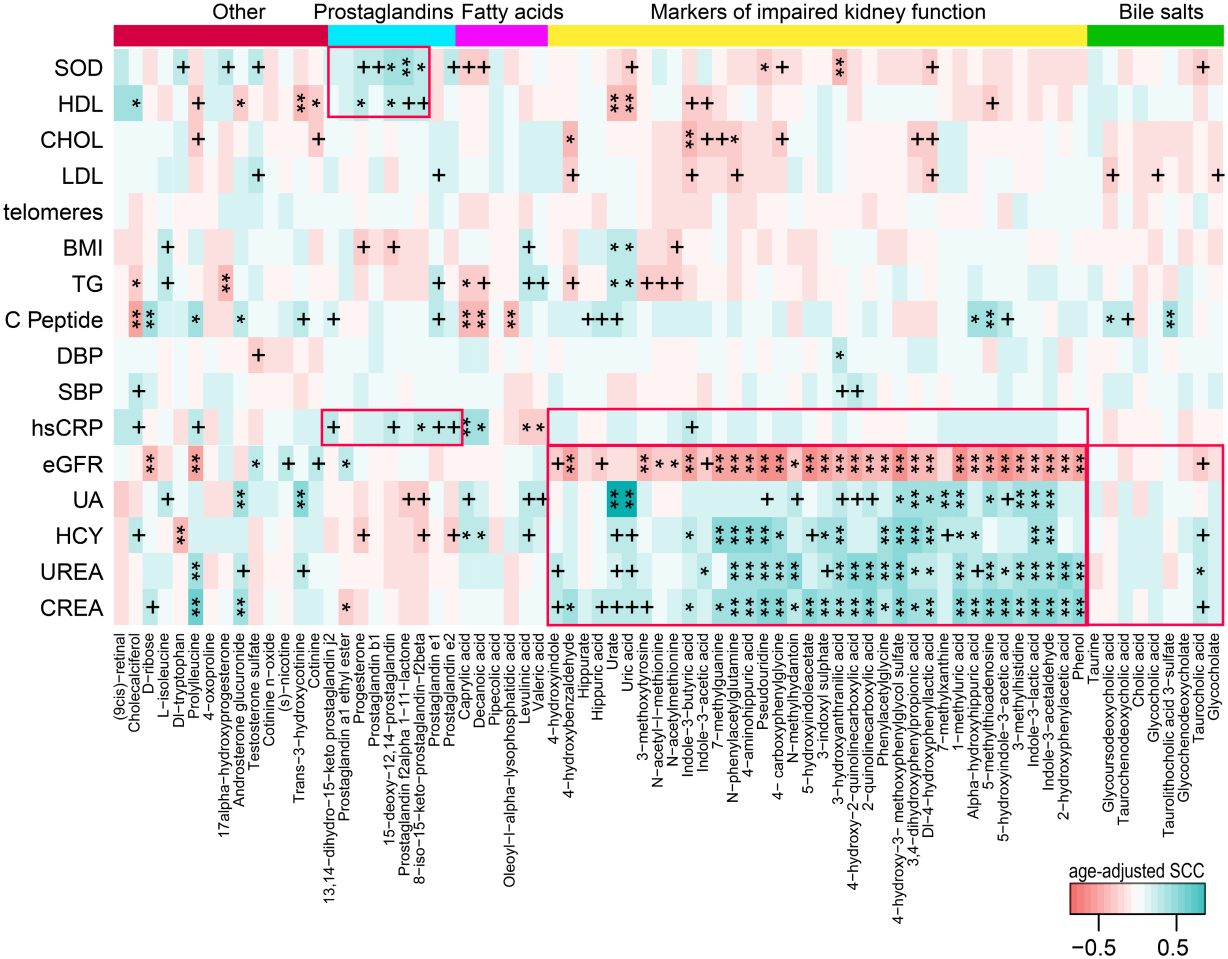
Covariation between serum metabolites and clinical parameters, as well as between serum uremic toxins and renal function indicators. The heatmap panel shows age‐adjusted Spearman correlation coefficients (SCC) between serum metabolites and clinical parameters. +*p* < 0.05; *p < 0.01; **p < 0.001. CHOL, total cholesterol; CREA, creatinine; DBP, diastolic blood pressure; eGFR, estimated glomerular filtration rate; HDL, high‐density lipoprotein; hsCRP, high‐sensitivity c‐reactive protein; LDL, low‐density lipoprotein; SBP, systolic pressure; SOD, superoxide dismutase; TG, triglycerides; UA, uric acid.

The serum metabolome also covaried with clinical parameters (Figure S5a), and age correlated significantly with the levels of 14 out of 26 clinical parameters (Figure S5b). Markedly lower levels of indicators of impaired renal function (CREA and UREA), SBP, homocysteine (HCY), and hsCRP were found in the older age groups. Importantly, after adjusting for the influence of age, the serum markers of impaired renal function were strongly positively associated with the renal function test indicators (CREA, UREA, and UA), as well as with HCY across the entire cohort, while being inversely associated with eGFR (Figure 3). This finding suggested that the levels of serum markers of impaired renal function share a close relationship with renal functional test indicators. In addition, the levels of taurocholic acid were weakly correlated with these indicators, while serum fatty acids and prostaglandins were significantly correlated with hsCRP (Figure 3). Previous reports have emphasized that uremic toxins, bile salts, and fatty acids are linked to the gut microbiota, and thus, microbiota‐derived uremic toxins and bile salts might lead to aggravated renal dysfunction (Wang et al., 2020).

### 3.4 The impact of the gut microbiota on serum metabolites increases with age

We next examined to what extent the gut microbiota might explain the serum metabolomics results with respect to different age groups using PERMANOVA. We observed an age‐related pattern in which the gut microbiota's effect sizes on serum metabolites accounted for 18.5%, 21.6%, 25%, and 26.6% of the serum metabolome variance in young‐to‐middle‐aged individuals, elderly individuals, nonagenarians, and centenarians, respectively (Figure S6, Table S8). Interestingly, the effect size of demographic (host property) and clinical parameters on the serum metabolome was significantly smaller than that of the gut microbiota across all age groups (5.5%, 6.8%, 6.8%, and 10.6% of the serum metabolome variance in young‐to‐middle‐aged adults, elderly individuals, nonagenarians and centenarians, respectively) (Figure S6, Table S8).

Age‐associated metabolites were further examined with regard to the functions of the gut microbiota. We found covariation between inferred gut microbiota functions and serum markers of impaired renal function (Figure S7). We hypothesized that the age‐associated enrichment of markers of impaired renal function might indicate gut microbiota‐mediated amino acid metabolism and microbial bile salt biosynthesis.

We then aimed to identify the bacterial species associated with markers of impaired renal function and bile salt alterations linked to aging, represented by p‐cresol, hippuric acid, 2‐oxindole, N‐phenylacetylglutamine, and phenol. Correlations were found between age‐related bacterial species and serum metabolites, particularly markers of impaired renal function (Figure S8). We identified genes encoding key synthetases that mediated the biosynthesis of these compounds (Figure S3, Table S9) and quantified their levels in the 14 MGSs with an age‐related upward trend, 8 MGSs with an age‐related downward trend, and 18 MGSs that had species‐ and/or genus‐level taxonomic assignment (Table S6). These analyses demonstrated that bacterial key synthetase genes encoding enzymes involved in indole, hippurate, and secondary bile acid synthesis (Table S9) and the microbial species harboring these genes were more abundant in the samples of older individuals than in younger individuals (Figure S9, Table S10).

**FIGURE 4:**
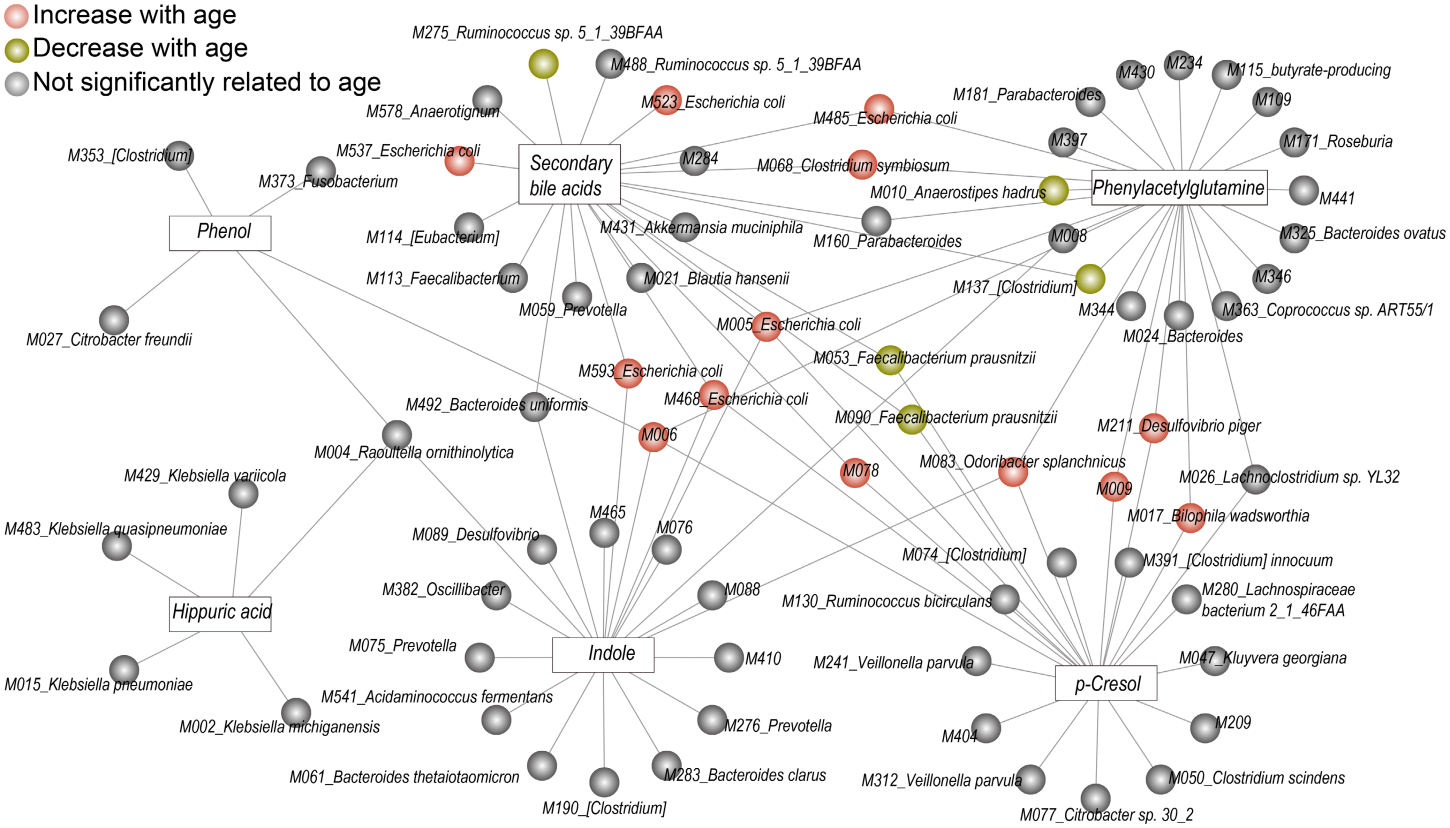
Age‐dependent relationships between the gut microbiota and serum metabolites. Network view of uremic toxins/bile acids and metagenomic species (MGSs). Squares represent uremic toxins or bile acids, and the surrounding connected circles represent the species that were used in the random forest models. Unclassified bacterial species are not included in the figure. SBAs, secondary bile acids.

Based on these findings, we next applied random forest models to estimate the correlation between uremic toxins (including 4‐methylphenol, 2‐oxindole, phenol, N‐phenylacetylglutamine, and hippuric acid) and serum bile acids (SBA; glycocholic acid, taurocholic acid, glycoursodeoxycholic acid, and taurochenodeoxycholic acid) and the abundance of synthetase‐encoding gut microbial species. Random forest models that maximized the predictive power of serum uremic toxins and bile salt concentrations identified 74 correlated MGSs (Figure 4, Table S11). The microbial species accounted for 22.05%, 9.41%, 0.63%, 33.25%, 4.38%, and 13.52% of the variance in 4‐methylphenol, 2‐oxindole, phenol, N‐phenylacetylglutamine, hippuric acid, and SBA concentrations, respectively, indicating that the corresponding species were the main contributors to the production of uremic toxins and bile salts. Significant correlations between bacteria and age were also observed in the case of *E. coli*, *F. prausnitzii*, *D. piger*, *O. splanchnicus*, *B. wadsworthia*, and *O. splanchnicus* (Table S6).

Based on the notion that the concentration of uremic toxins and bile salts might be influenced independently through other pathways (e.g., metabolite transport), we extended the random forest models to include species that lacked synthetases, whereby the updated model could account for an additional ~18.59% of the variance (Figure S10, Table S12). Although identified MGSs based on current methods might not capture the entire profile of the gut microbiota, high correlations among the gut microbiota, renal toxins, and bile salts were still found.

Some of the gut microbial species that were linked to uremic toxins or bile salts were also correlated with the renal functional test indicators (Figure S11). In particular, a high proportion of the variance (an average of 21.76%) of eGFR, CREA, UREA, and UA was explained by the abundances of *E. coli*, *Klebsiella michiganensis*, *Klebsiella quasipneumoniae* and *Klebsiella pneumoniae* (Figure S11, Table S13). In addition, *Veillonella parvula* and [*Clostridium*] spp. were significantly correlated with eGFR, hsCRP, and CREA. *Adlercreutzia equolifaciens* was significantly correlated with hsCRP and UA (Figure S11, Table S13). Previous reports have emphasized that *Klebsiella* spp., *E. coli*, and *V. parvula* are linked to aging (Zhang et al., 2021).

### 3.5 Microbiota‐metabolite interactions in aging and FMT experiments

**FIGURE 5:**
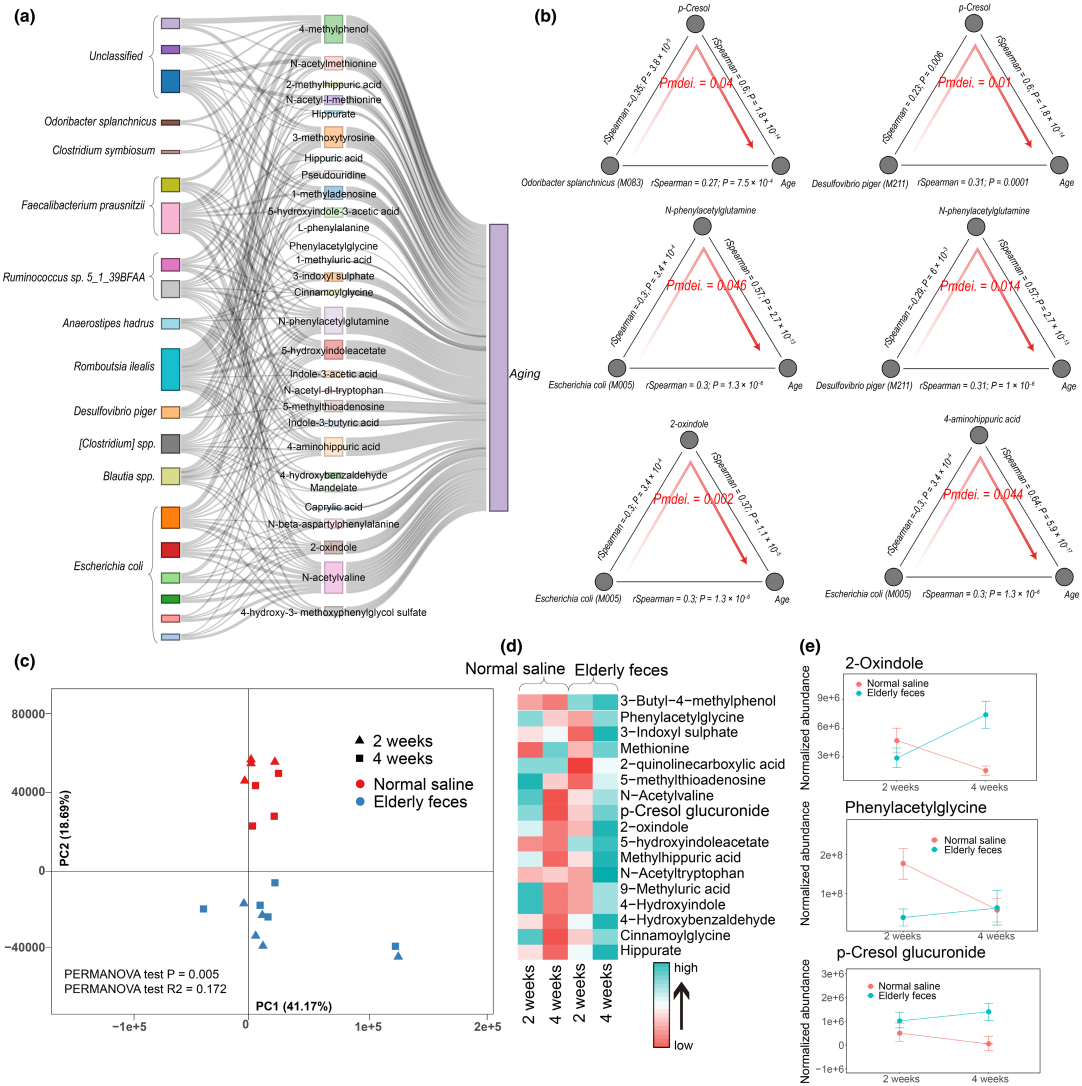
Mediation analysis and FMT experiments identify linkages between the gut microbiome, metabolites, and aging. (a) Parallel coordinates chart showing the 168 mediation effects of serum markers of impaired renal functions that were significant at *p* < 0.05. Shown are markers of impaired renal functions (left), microbial factors (middle) and age (right). The curved lines connecting the panels indicate the mediation effects, with colors corresponding to different metabolites and microbes. (b) Analysis of the effect of *O. splanchnicus*, *D. piger* and *E. coli* on aging as mediated by hippuric acid, N‐phenylacetylglutamine, 2‐oxindole, and 4‐aminohippuric acid. (c) PCA shows a clear separation between the serum metabolome of mice gavaged with saline and those gavaged with feces from elderly humans. (d) Differences in metabolites related to renal function in the serum metabolome between mice gavaged with saline and those gavaged with feces from elderly humans, as well as changes in their metabolite levels at different time points (*p* < 0.1). (e) Changes at 2 and 4 weeks in 2‐oxindole, phenylacetylglycine, and p‐cresol glucuronide in mice gavaged with saline and those gavaged with feces from elderly humans (*p* < 0.1).

Next, we carried out a mediation analysis to investigate the links among the gut microbiota, serum metabolites, and aging. For the 22 gut microbial features that were associated with both serum metabolites and aging (FDR < 0.05), we applied a bidirectional mediation analysis to evaluate whether the effect of the gut microbiota on aging is mediated via serum metabolites. This approach established 524 mediation linkages for the impact of the gut microbiota on serum metabolites through aging (*p* < 0.05 and Pinverse‐mediation >0.05; Figure 5a, Table S14). Most of these linkages were related to the impact of *E. coli*, *R. ilealis*, and *F. prausnitzii* on N‐phenylacetylglutamine and 4‐methylphenol (Figure 5a).

*E. coli*, harboring genes encoding indole and phenylacetylglutamine synthetases, can convert tryptophan and phenylalanine into indole or phenylacetylglutamic acid (Table S14). We observed that the effect of *E. coli* on aging is mediated via N‐phenylacetylglutamine, 2‐oxindole, and 4‐aminohippuric acid (*P*_mediation_ = 0.046, 0.002, and 0.044; Figure 5b). We also observed that the effect of *O. splanchnicus* and *D. piger* on aging is mediated via p‐cresol (*P*_mediation_ = 0.004, 0.001, Figure 5b). In addition, the effect of *D. piger* on aging is mediated via N‐phenylacetylglutamine (*P*_mediation_ = 0.014, Figure 5b).

To verify our results, we transplanted the fresh gut microbiota from 20 elderly donors into mice treated with antibiotics. Compared with mice gavaged with physiological saline, those receiving the elderly microbiota exhibited significant differences in the gut microbiota and serum metabolome (Figure 5c, Figure S12a). Through analyses of the gut microbiota and serum metabolome at 2 weeks and 4 weeks, the abundances of *Odoribacter* and *Desulfovibrio* in the mice receiving the elderly microbiota were significantly elevated after 4 weeks (Figure S12b). Concomitantly, 17 markers related to impaired renal function were also significantly elevated after 4 weeks, especially 2‐oxindole, p‐cresol glucuronide, and phenylacetylglycine (Figure 5d,e). Overall, these results demonstrate that the gut microbiota of elderly individuals can regulate markers related to impaired renal function in the serum.

### 3.6 Specific patterns associated with aging in long‐living individuals

**FIGURE 6:**
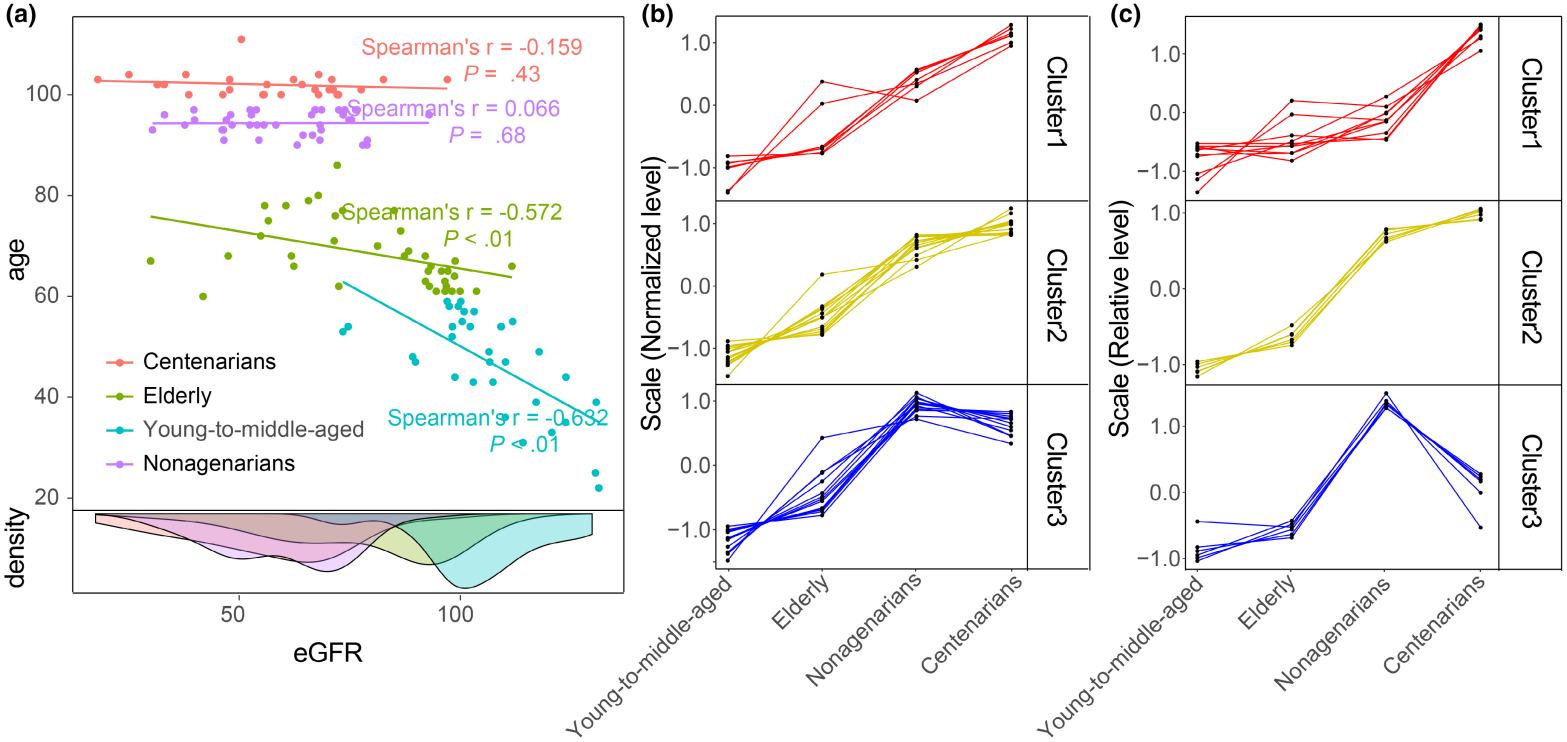
Different fluctuation trend patterns of age‐related markers related to impaired renal function and the age‐related gut microbiota in the oldest individuals. (a) The relationship between eGFR and age in different age groups. (b, c) Different fluctuation trend patterns of age‐related markers related to impaired renal function (b) and age‐related gut microbiota (c). Cluster1: metabolites different in centenarians ≥1.2* metabolites in nonagenarians, Cluster2: metabolites different in centenarians <1.2* metabolites in nonagenarians and metabolites in centenarians >metabolites in nonagenarians, Cluster3: metabolites different in centenarians ≤metabolites in nonagenarians.

Centenarians, as a model of extreme aging, may provide information on the relationships among the gut microbiota, healthy aging, and longevity. Here, we found that the four age groups differed with respect to eGFR, particularly nonagenarians and centenarians (Figure 6a). Although the eGFR in these long‐living individuals (nonagenarians and centenarians) was significantly (Wilcoxon rank‐sum test, *p* < 0.001) lower than that in younger individuals (young‐middle‐aged and elderly), gradually flattened slopes of eGFR decline were observed in the case of extreme aging. Since a higher GFR is associated with healthy aging (Eriksen et al., 2020), we used eGFR as an indicator to stratify the long‐living individuals and younger individuals (low‐eGFR group and high‐eGFR group). We found that 24.2% (31/128) and 41.4% (53/128) of age‐related metabolites were significantly associated (FDR *q* < 0.05) with the eGFR in long‐living individuals and younger individuals, respectively (Table S16). In particular, 16 and 25 markers of renal impairment were significantly correlated with the eGFR in long‐living and young individuals, respectively, and were enriched in the low‐eGFR group (Figure S13, Table S16), indicating that longevity might depend on the maintenance of renal function. In addition, no significant association (FDR *q* > 0.05) between the gut microbiota and eGFR was found in long‐living or younger individuals.

To interpret the finding of “delayed aging of kidneys” in long‐living individuals, we classified the fluctuation trends of the levels of uremic toxins according to age into three distinct clusters (Figure 6b): cluster 1, with levels of markers of impaired kidney function increasing steadily with age, mainly including hippuric acid, phenylacetylglutamine, acetylated methionine and valine, and 3‐methylhistidine; cluster 2, with levels of markers of impaired kidney function increasing slowly in centenarians, mainly including derivatives of hippuric acid, p‐cresol, and phenylacetylglycine; cluster 3, with levels of markers of impaired kidney function decreasing in centenarians compared with nonagenarians, mainly including indole derivatives, phenylalanine and phenylalanine derivatives. The cluster 3 trend of markers of impaired kidney function levels was of particular interest, as the derivatives of indole may promote cellular senescence and premature aging through toxic alterations in the internal milieu (Adijiang et al., 2010; Stenvinkel & Larsson, 2013).

Similar trends for specific gut microbes underpinned the role of the gut microbiota in delaying the accumulation of markers of impaired kidney function in centenarians. Bacteria with increased, relative abundances in the older individuals could also be classified into three clusters (Figure 6c): cluster 1 mainly included *D. piger*, *Alistipes finegoldii*, and *C. symbiosum*; cluster 2 mainly included *E. coli*; and Cluster 3 mainly included *E. cloacae*. The covariation of specific gut microbes (*E. coli* and *E. cloacae*) and specific uremic toxins (derivatives of hippuric acid, p‐cresol, and indole) indicated the potential impact of the gut microbiota on renal function. Notably, *E. coli* and *E. cloacae* have been reported to be involved in the production of the precursors of uremic toxins in the intestine (Kikuchi et al., 2017).

Our results revealing the interplay between the gut microbiota and the serum metabolome suggest that delayed renal aging in long‐living individuals may reflect diminished accumulation of certain markers of impaired kidney function.

## 4 DISCUSSION

Based on residents from a Chinese longevity county, with long‐living individuals (nonagenarians and centenarians) as healthy aging controls, this study aimed to examine the possible relationship between renal function and age‐associated alterations in the human gut microbiota and serum metabolome using an integrated omics approach. Our results indicated that the effect of the gut microbiota on serum metabolites increased with age and that many age‐associated gut microbes (*E. coli*, *O. splanchnicus*, and *D. piger* in particular) and serum metabolites, including markers of impaired renal function and bile acids, were highly correlated. The relationships between renal functions (eGFR, CREA, UREA, and UA), serum metabolites, and the gut microbiota further indicated a possible impact of the gut microbiota in the aging process. Through mediation analyses, we revealed putative causal relationships among the gut microbiota (*E. coli*, *O. splanchnicus*, and *D. piger*), markers related to impaired renal function (p‐cresol, N‐phenylacetylglutamine, 2‐oxindole, and 4‐aminohippuric acid) and age. The FMT experiment demonstrated that the feces of elderly individuals could influence markers related to impaired renal function in the serum. Thus, this study not only revealed changes in the serum metabolome and the gut microbiota in the process of aging but also indicated a route by which the gut microbiota affects aging indirectly through its effect on renal function via the production of metabolites associated with impaired renal function.

Unlike the previously reported (Wu et al., 2019; Zhang et al., 2021) cross‐sectional study of aging, we adopted a multiomics approach and combined the correlation between gut microbiota and serum metabolome to analyze the aging process in more detail. Our analyses showed that the effect sizes of the gut microbiota on serum metabolites increased with age. It has been reported that the intestinal mucosal barrier does not deteriorate with age per se (Saltzman et al., 1995; Valentini et al., 2014), but low‐grade chronic inflammation and mild diseases may impair the intestinal barrier, potentially indicative of chronic renal disease (CKD) (Meijers et al., 2018). Under normal circumstances, the kidneys excrete metabolites that serve as markers of impaired renal functions in urine. Most renal problems are caused by the gradual loss of glomerular filtration function, whereby transport of potentially toxic compounds from the blood to the urine is impaired, leading to their accumulation in the body (Vanholder et al., 2001). The accumulation of these compounds has a negative impact on many body functions and leads to gradual endogenous poisoning (Vanholder et al., 2008). The levels of creatinine and pseudouridine in the urine metabolome of healthy elderly people were reported to be lower than that those of young people (Chen et al., 2020), which is consistent with the notion that renal problems can occur with age. With increasing age, in general, glomerular filtration function decreases, which leads to the failure to effectively remove metabolites associated with impaired renal function from the blood, accompanied by a decrease in these metabolites in the urine.

Consistent with previous gut microbiota studies (Rampelli et al., 2020; Wu et al., 2019), we observed age‐dependent changes in the abundances of *E. coli*, *F. prausnitzii*, and *O. splanchnicus*. *E. coli* was one of the most noticeable species that changed in abundance with age and was predicted to be associated with the production of several metabolite markers of impaired renal functions, including indole, p‐cresol, and phenylacetylglutamine. In keeping with our results, *E. coli* has been reported to be involved in the degradation of tryptophan into indole or p‐cresol (Yanofsky, 2007). Other species associated with the production of important markers of impaired renal function included *D. piger* and *O. splanchnicus*, identified as participating in the production of phenylacetylglutamine, indole, and p‐cresol. Notably, *D. piger*, as a potentially “harmful” bacterium, was recently reported to synthesize more toxins than it can degrade (Popkov et al., 2022). *O. splanchnicus* is capable of generating all four protein‐bound uremic toxin precursor metabolites under anaerobic conditions (Gryp et al., 2020). Our work thus suggests that the metabolic alterations in the intestinal tract contributed significantly to the accumulation of uremic toxins in serum with age, and species producing these marker metabolites clearly contribute significantly to the accumulation of gut‐derived uremic toxins with aging.

The impact of aging on the serum metabolome is obviously influenced by diet, but the effect of diet is difficult to disentangle. On the one hand, the dietary habits of people of different ages are based on preferences or availability, which are particularly likely to differ between urban and rural areas. Our research was based on household surveys and cluster sampling within a relatively small area, so the dietary habits, diet structure, and physical activity habits of all participants were relatively similar and homogeneous. On the contrary, with increasing age, degenerative changes occur in the digestive system, which may lead to difference in dietary preferences, thus eliciting changes in gut microbes and metabolites. This physiological change is difficult to eliminate. In addition, regarding internal factors, aging is also associated with a decline in metabolic capacity, specifically reflected in the weakening of anabolism and the increase in catabolism, and elderly individuals often present a relatively malnourished state compared to middle‐aged individuals. Thus, age‐dependent differences in diets, lifestyle, and genetics altogether may lead to changes in the gut microbiota and thus changes in the production of metabolites that may affect renal function.

The extent to which changes in the gut microbiota and kidney function are causally linked needs further clarification. FMT experiments have demonstrated that the feces from elderly individuals could elevate relevant markers related to kidney function in serum, but tracing these changes back to the changes in key bacteria and corresponding metabolites might fully explain the importance of the gut microbiota in the aging process. In fact, a previous study has emphasized the relevance of renal function with regard to premature aging (Kooman et al., 2014). As people age, progressive declines in multiorgan functions are inevitable (López‐Otín et al., 2013). The decline in renal function might weaken the detoxification capacity of the aged body and may accelerate the aging process (Weinstein & Anderson, 2010). Severely impaired renal function may lead to fatal conditions, such as CKD (Liyanage et al., 2015). The progression of renal function deterioration to CKD and its comorbidities are closely related to the accumulation of toxic metabolites in the blood (Zhang et al., 2012) with numerous marker metabolites being produced by the gut microbiota via the conversion of diet‐derived AAAs and polyphenols (Wang et al., 2020). We, therefore, propose that age‐dependent changes in the composition and functional potential of the gut microbiota contribute, at least in part, to the aging process and that maintenance of gut microbiota homeostasis and kidney health may enhance physical fitness in long‐living individuals. Future research using targeted analysis of serum metabolites will be necessary to further our understanding of the importance of perturbations of the gut microbiota and serum metabolome in aging and longevity. To better study the effect of the gut microbiota in the process of aging, examination of the fecal metabolome and transcriptome of the gut'microbiota will be needed, adding crucial functional information.

Our findings contribute to the growing body of evidence on the relationship between gut microbiota and aging. while a recent study also address the potential association of gut microbiota with longevity with 16S sequencing and found centenarians were reflected by the gut microbiome with youth‐associated signatures (Pang et al., 2023), our study differs from it in several ways. Firstly, we just focused on a longevity county approved by the Chinese Gerontology Society, potentially reducing the confounding of genetic background and lifestyle such as diet; and validated with external cohorts. Secondly, our research took a multiomics approach by comprehensively examining profiling of the metagenome and serum metabolome. This enabled us to investigate the microbial producers of these metabolites and their changes throughout the aging process, thereby enhancing our understanding of aging. Lastly, our study has uncovered unique microbial signatures and metabolites, as well as metabolic pathways that have not been previously reported in aging studies. These findings not only advance our knowledge of the complex mechanisms underlying aging but also offer potential new targets for interventions aimed at promoting healthy aging and longevity.

In summary, this study revealed important characteristics of the gut microbiota and serum metabolome during aging and how age‐related changes in the gut microbiota are associated with an accumulation of distinct markers of impaired renal function in the blood. In particular, the accumulation of markers of impaired renal functions and a reduction in renal function may accelerate the aging process, emphasizing the importance of gut microbiota alterations and markers of impaired renal function in healthy aging.

## AUTHOR CONTRIBUTIONS

Liang Sun, Ze Yang, and Chao Nie designed this study; Caiyou Hu, Rui Li, Ying Zhang, Chen Chen, Haiyun Guo, Yuan Lv, Qizhi Cao, Yanan Sun, Zezhi Huang, Zhu Wu, Zhen Jin, Junchun Li, Ranhui Gong, Jian Li, Wenbin Xue, Ruiyue Yang, Guofang Pang, Jun Dong, Shuqin Sun, Jianmin Zhang, Yuzhe Sun, Juan Shen, Fan Yang, Benjing He, Cheng Zhao, Ninghu Li, and Qinghua Liang coordinated volunteer recruitment; Yuzhe Sun, Hengshuo Liu, Hefu Zhen, Mingyan Fang, Zhihua Chen, Mingrong Zhang, and Jie Ruan performed DNA extraction, experimental method testing and optimization, and sequencing library construction; Liang Sun, Zhiming Li and Jiahong Ding, Haorong Lu, Yan Li performed data analysis and interpretation; Liang Sun, Ze Yang, Chao Nie, and Caiyou Hu supervised the whole project; Zhiming Li, Liang Sun, Jiahong Ding, Qi Zhou, Yong Duan, Huan Gong, Yuan Lv, Tao Li, Liang Xiao, and Peng Zhao drafted the manuscript, which was substantially revised by Karsten Kristiansen, Chao Nie, and Liang Sun; Karsten Kristiansen, Huanming Yang, Susanne Brix, Jian Li, Chao Nie, Huijue Jia, Yong Hou, Shida Zhu, Jianping Cai, Jian Wang, and Xun Xu facilitated scientific discussion and gave suggestions; and all the authors have read and approved the manuscript.

## CONFLICT OF INTEREST STATEMENT

The authors disclose no conflicts.

## FUNDING INFORMATION

This work was financially supported by the National Scientific Foundation of P. R. China (U23A20470, 82260289, 91849132, and 81571385), the CAMS Innovation Fund for Medical Sciences (2021‐I2M‐1‐050), the National Key Research and Development Program of China (2021YFE0111800, 2023YFC3603300 and 2018YFC2000400), National High Level Hospital Clinical Research Funding (BJ‐2023‐168 and BJ‐2023‐075), the Science and Technology Innovation 2030 Major Projects (2022ZD0211600), the Priority Union Foundation of Yunnan Provincial Science and Technology Department (202001AY070001‐011), and the Beijing Hospital Nova Project (BJ‐2018‐139).

## Supporting information


Table S1.
Table S2.
Table S3.
Table S4.
Table S5.
Table S6.
Table S7.
Table S8.
Table S9.
Table S10.
Table S11.
Table S12.
Table S13.
Table S14.
Table S15.
Table S16.

Figure S1.
**Figure S2**.
**Figure S3**.
**Figure S4**.
**Figure S5**.
**Figure S6**.
**Figure S7**.
**Figure S8**.
Figure S9.
Figure S10.
Figure S11.
Figure S12.
Figure S13.

## Data Availability

Data have been deposited in the CNSA (https://db.cngb.org/cnsa/) of CNGBdb with accession number CNP0000634. For statistical and metagenomic analyses codes are available on https://github.com/lizhiming11/aging‐omics. Other data that support the findings of this study are available from the corresponding authors upon request.
