# Supplementary material for: Age‐dependent changes in the gut microbiota and serum metabolome correlate with renal function and human aging
Source: Aging Cell. 2023 Nov 27;22(12):e14028. doi: 10.1111/acel.14028 (PMC10726799; doi:10.1111/acel.14028)
Supplement: Supplementary file 2 — Figure S1. Figure S2. Figure S3. Figure S4. Figure S5. Figure S6. Figure S7. Figure S8. Figure S9. Figure S10. Figure S11. Figure S12. Figure S13. [file ACEL-22-e14028-s001.docx]

**Supplementary Materials for**

**Age-dependent changes in the gut microbiota and serum metabolome correlate with renal function and human aging**

**This PDF file includes:**

Fig. S1 to S13

Captions for Table S1-16

**Other Supplementary Materials for this manuscript include the following:**

Supplementary Table S1-16 (Excel)

**Supplementary Figures （Figure S1-13）**

**
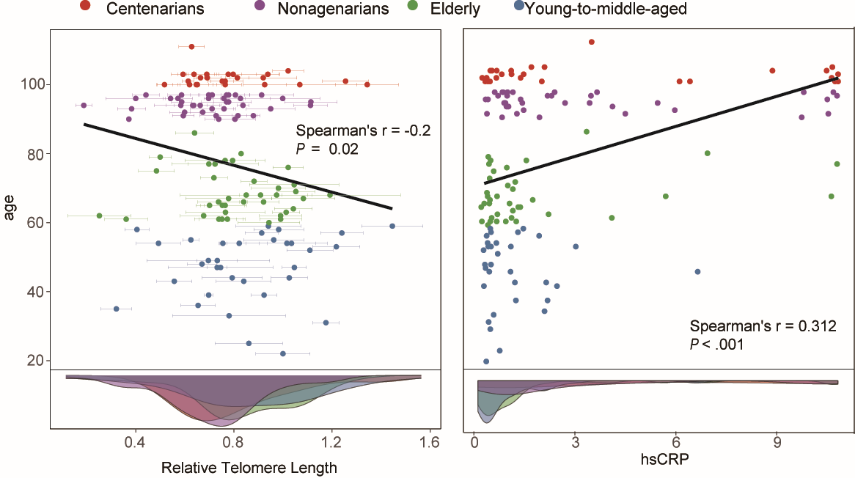
**

**Figure S1. Correlations between age and relative telomere length, and age and high-sensitivity C-reactive protein (hsCRP).**

**
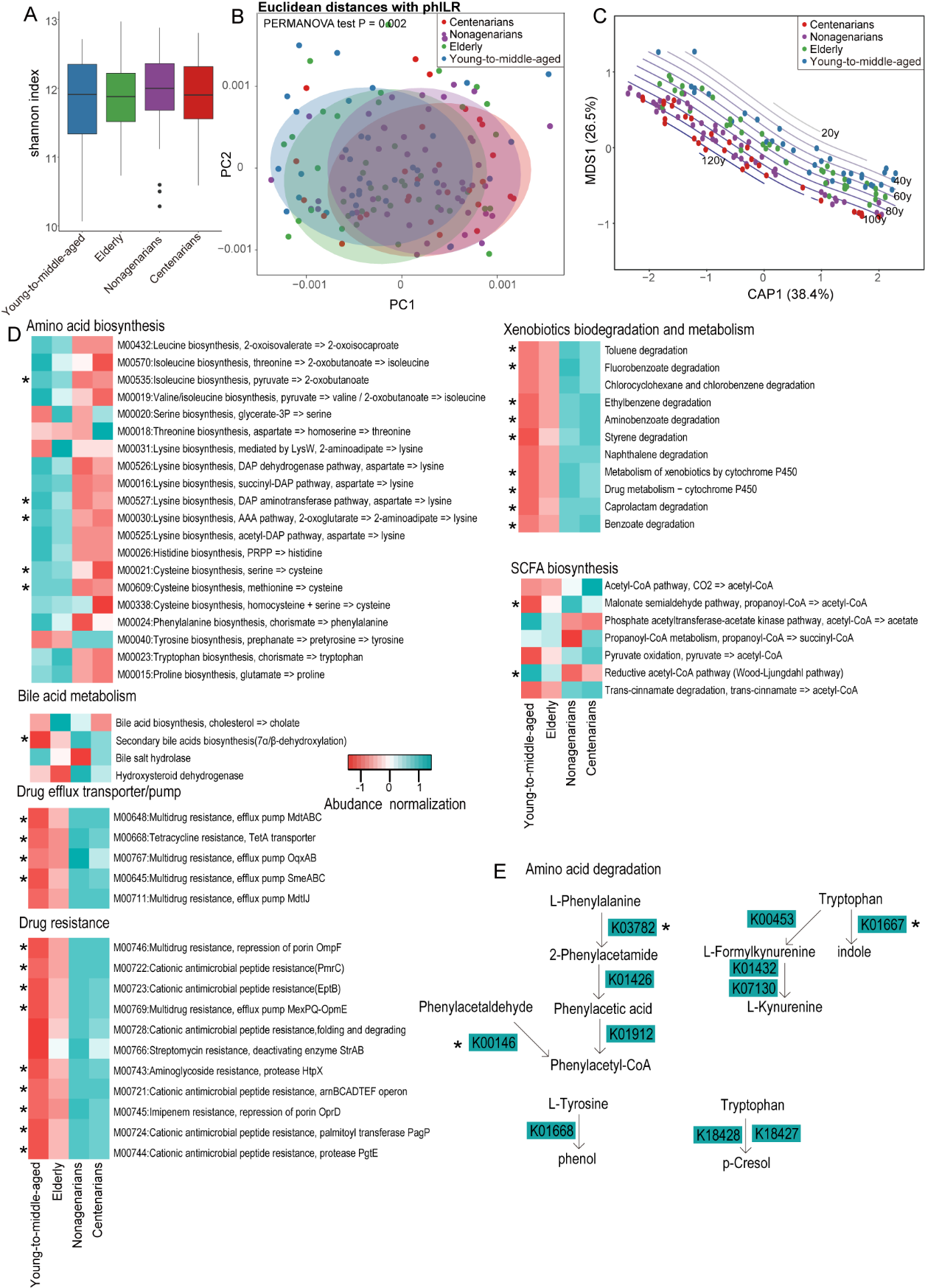
**

**Figure S2. Alteration of gut microbial functions in young-to-middle-aged adults, elderly individuals, nonagenarians, and centenarians.** (*A*) Box plots showing the gene-based alpha-diversity (Shannon index) in young-to-middle-aged adults (n = 35), elderly (n = 41), nonagenarians (n = 46) and centenarians (n = 29). (*B*) PhILR analysis of bacterial species (MGS) of young-to-middle-aged adults, elderly, nonagenarians and centenarians. (*C*) Gut microbial function dissimilarity-based redundancy analysis (dbRDA) of young-to-middle-aged adults, elderly, nonagenarians and centenarians. (*D*) Alterations in gut microbial pathways and functional modules. Red: the deeper the color, the lower the relative abundance; Cyan: the darker the color, the higher the relative abundance. The significance levels (FDR) in the Kruskal-Wallis are denoted as follows: *, *q* < 0.05. (*E*) Reaction steps for the synthesis of indole, p-Cresol, phenol and Phenylacetyl-CoA. Green represents that the relative abundance of this function increases with age. *: Spearman correlation with age *q* <0.05.


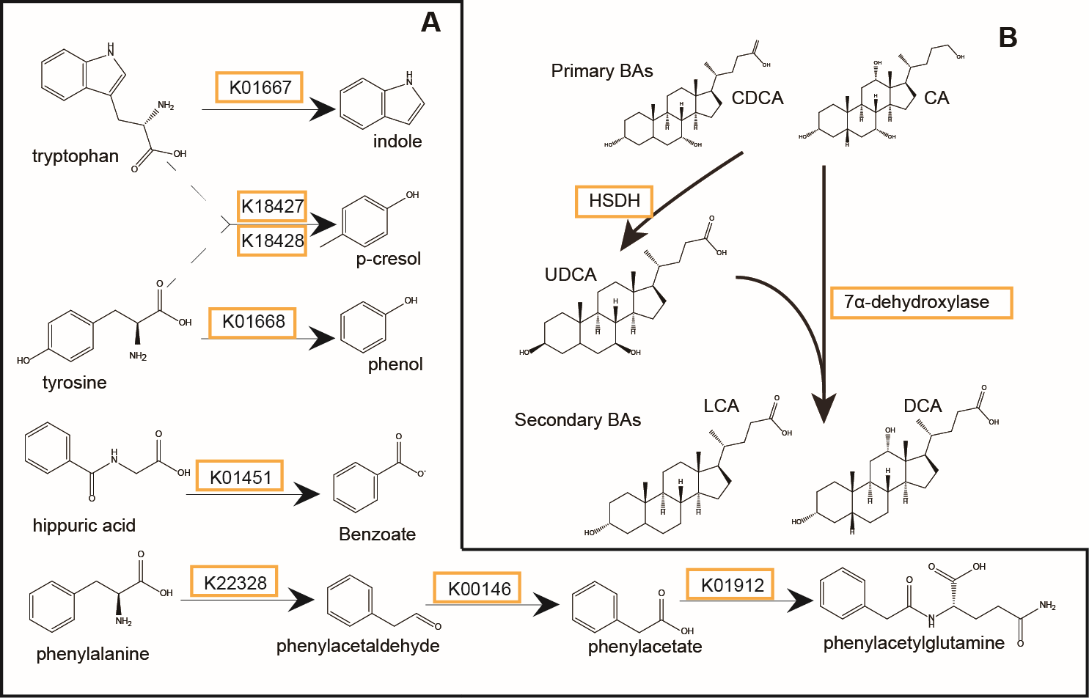


**Figure S3.** **Schematic representation of the metabolic pathways of serum uremic toxins and bile acids associated with the gut microbiota.** (*A*) Production of serum uremic toxins by gut microbiota by degrading diet-originated aromatic amino acids, polyphenols and choline. (*B*) Metabolism of bile acids. Orange boxes highlight the key enzymes encoded by gut microbes, and details are shown in Supplementary Table 8.


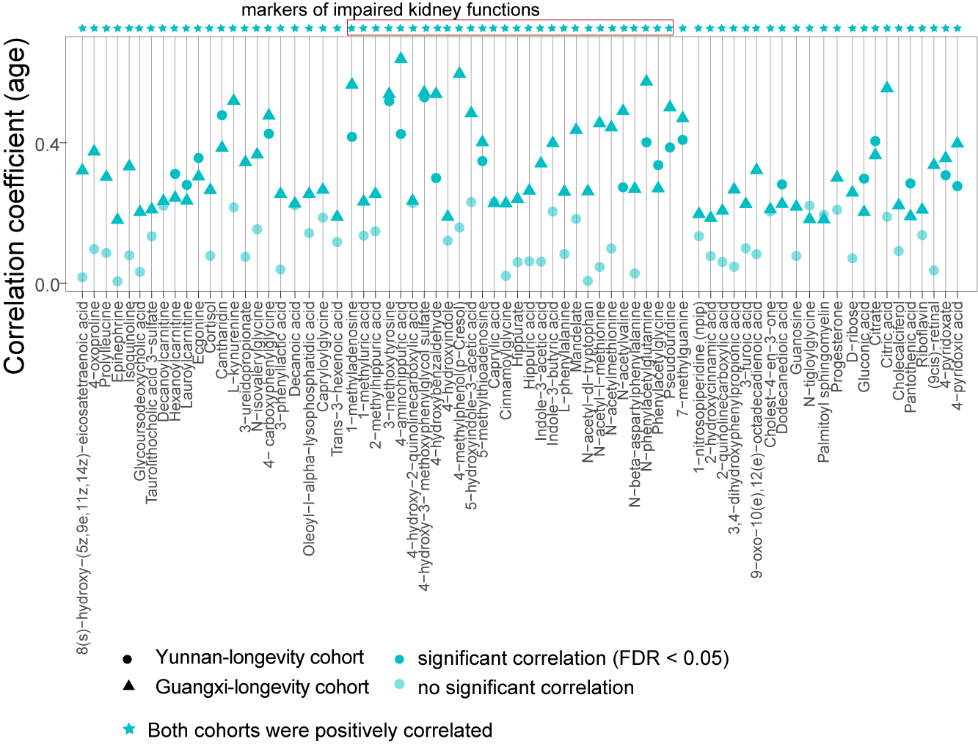


**Figure S4.** **Age related serum metabolites in the Guangxi longevity cohort and the Yunnan aging cohort.**


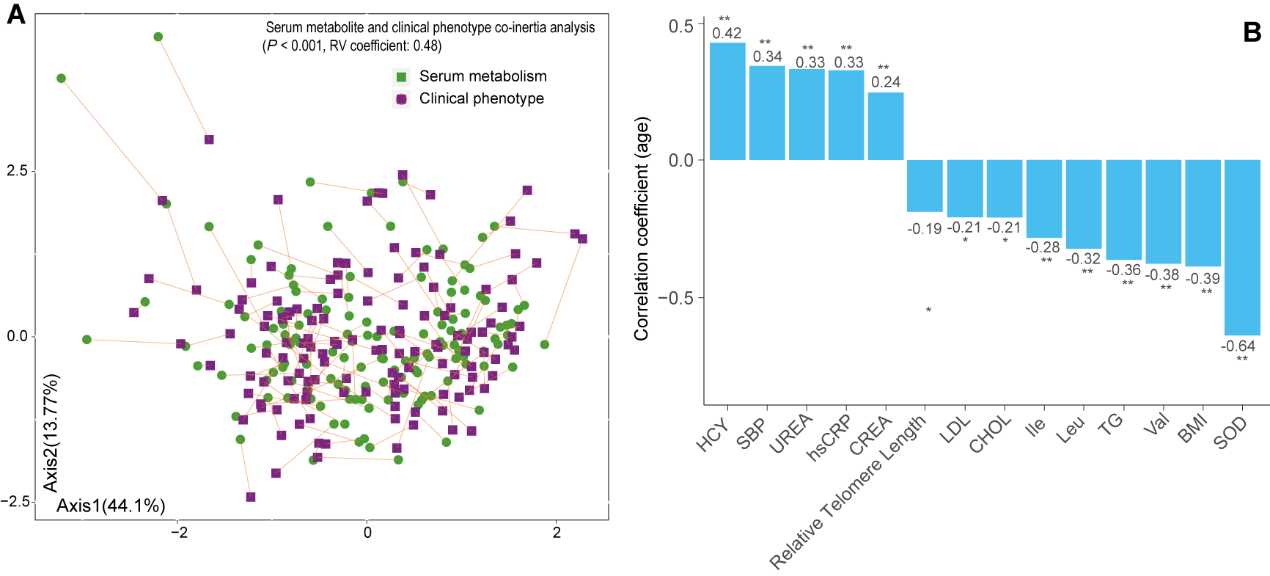


**Figure S5.** **Co-variation between serum metabolome and host phenotype.** (*A*) Co-inertia analysis (CIA) of the relationships between serum metabolites and clinical phenotypes. Each sample is represented by a purple square (serum metabolites) or a green circle (clinical parameters), and lines represent sample projections. (*B*) Bar chart depicting clinical parameters with age by the Spearman correlation. The number on the bar chart represents the Spearman correlation coefficient, and the significance levels in the Spearman correlation are denoted as follows: *, *P* < 0.05; **, *P* < 0.01. Abbreviations: HCY: homocysteine, hsCRP: high sensitivity c-reactive protein, TG: triglycerides, CHOL: total cholesterol, CREA: creatinine, UA: uric acid, LDL: low-density lipoprotein, HDL: high-density lipoprotein, DBP: diastolic blood pressure, SBP: systolic pressure, SOD: superoxide dismutase.


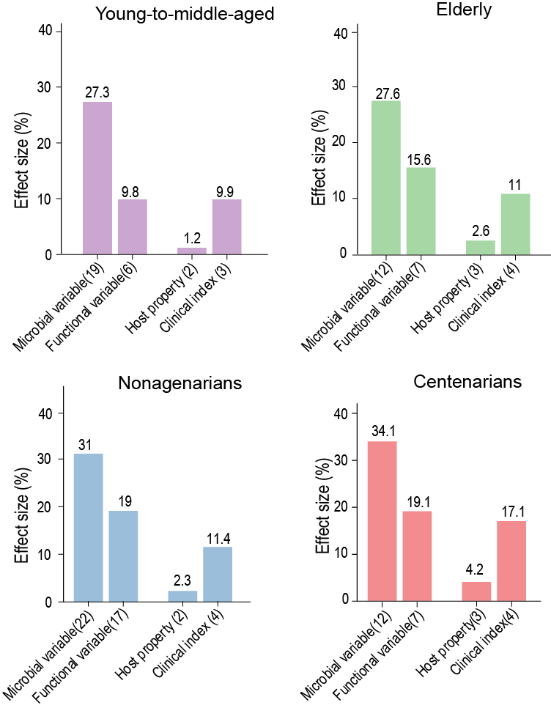


**Figure S6. Age-dependent associations between the gut microbiota and host serum metabolome.** The percentage of the total variation in the serum metabolomes of young-to-middle-aged individuals, elderly individuals, nonagenarians, and centenarians explained by the gut microbiome and host phenome. To calculate the effect size, a set of nonredundant covariates were selected from the gut microbiome (including microbial and functional variables) or host phenome (including host phenotypes and clinical parameters) by a stepwise PERMANOVA analysis. The number of nonredundant covariates is shown in parentheses.


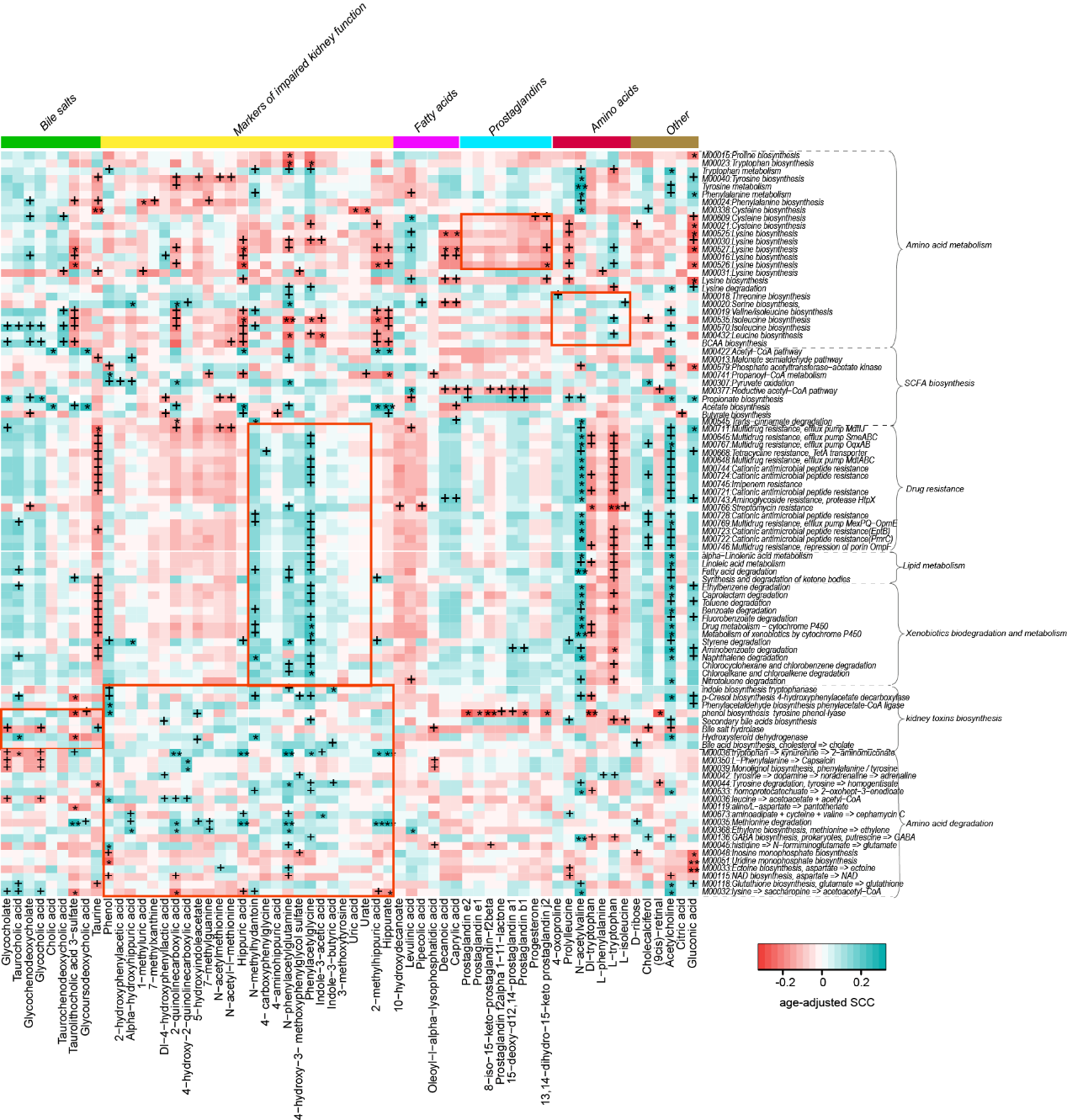


**Figure S7.** **Correlation between serum metabolites and gut microbial functions.** The heatmap panel shows the Spearman correlation coefficients between age-adjusted functional modules and serum metabolites. The significance levels in the correlation test are denoted as follows: +, *P* < 0.05; *, *P* < 0.01; **, *P* < 0.001.


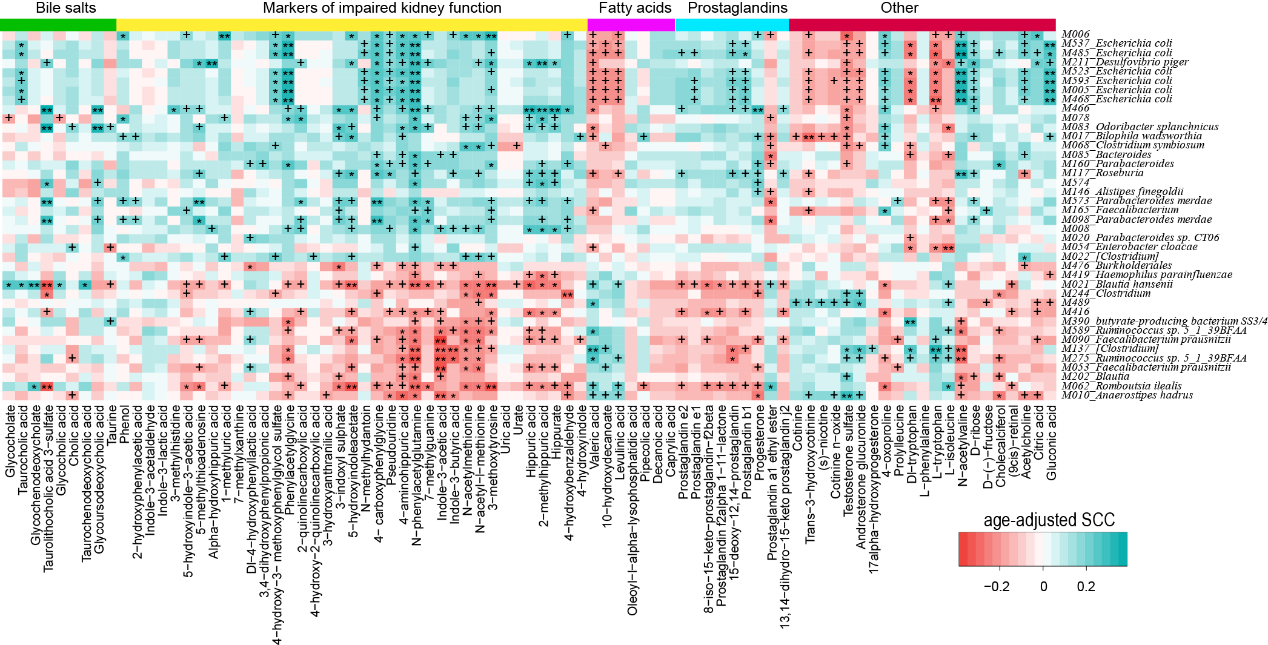


**Figure S8.** **Correlation between serum metabolites and gut microbiota.** The heatmap panel shows the Spearman correlation coefficients between age-adjusted age-related gut microbiota and age-related serum metabolites. The significance levels in the correlation test are denoted as follows: +, *P* < 0.05; *, *P* < 0.01; **, *P* < 0.001.


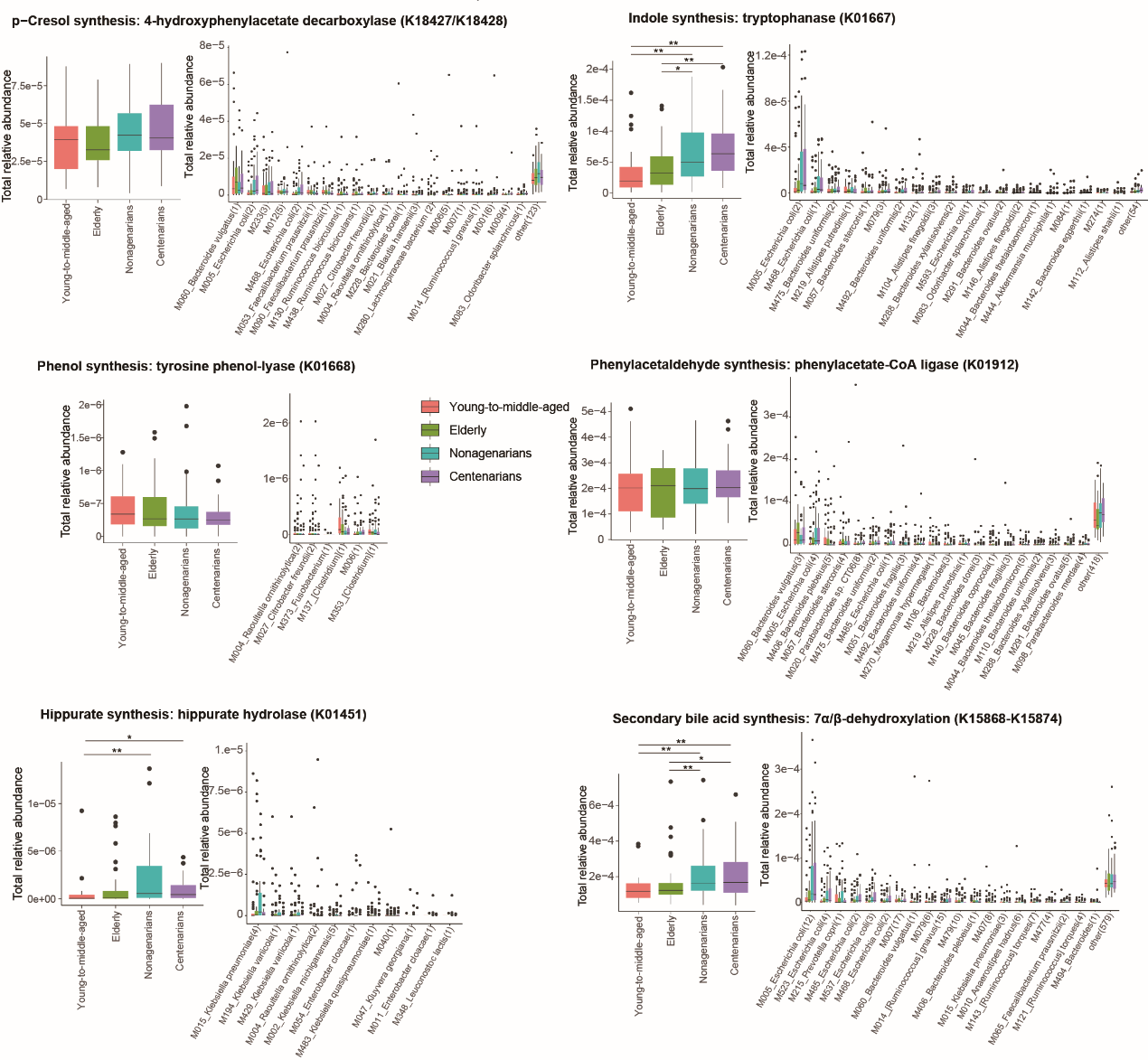


**Figure S9.** **Distribution of the key synthetases involved in the biosynthesis of uremic toxins and bile salts.** Relative abundances of key synthetase-encoding catalog genes in all samples were shown. The number of synthetase-encoding genes in each MGS is shown in parentheses. Boxes represent the interquartile range between the first and third quartiles and median (internal line). Whiskers denote the lowest and highest values within 1.5 times the range of the first and third quartiles, and circles represent outliers beyond the whiskers.


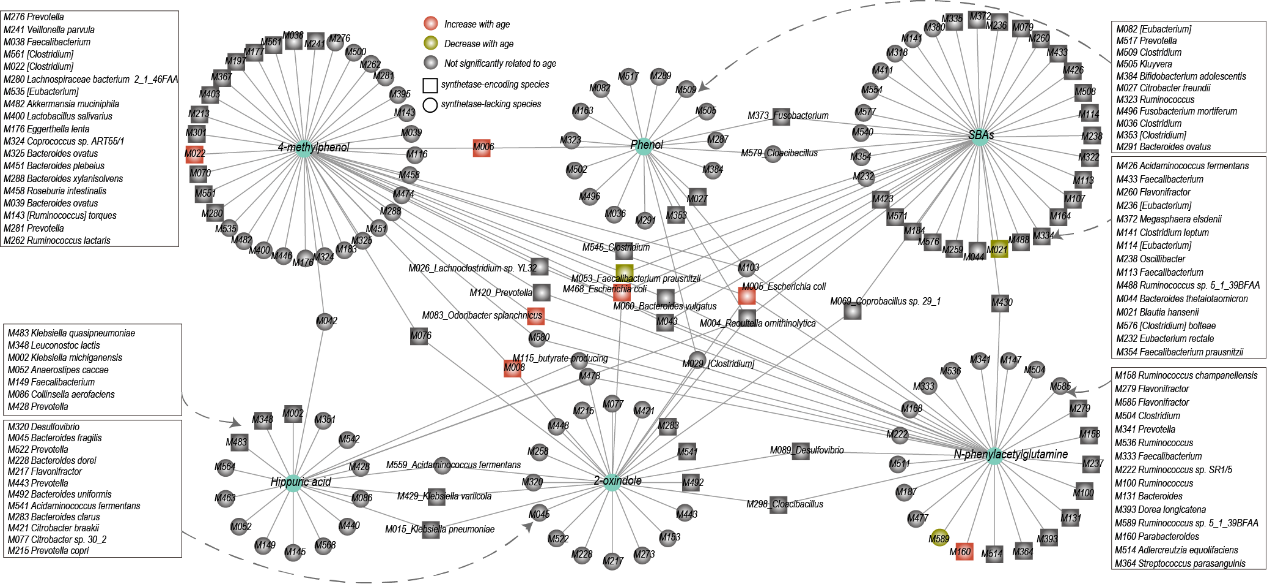


**Figure S10.** **Network view of the uremic toxins/bile salts and the MGSs used to construct predictive models.** Central boxes represent 4-methylphenol, phenol, bile salts, hippuric acid, 2-oxindole, and N-phenylacetylglutamine in serum. Positive and negative correlations of MGSs with age are highlighted in red and green, respectively; MGSs that are not significantly correlated with age are indicated in grey. The MGSs without taxonomic annotation are not included in the lists.


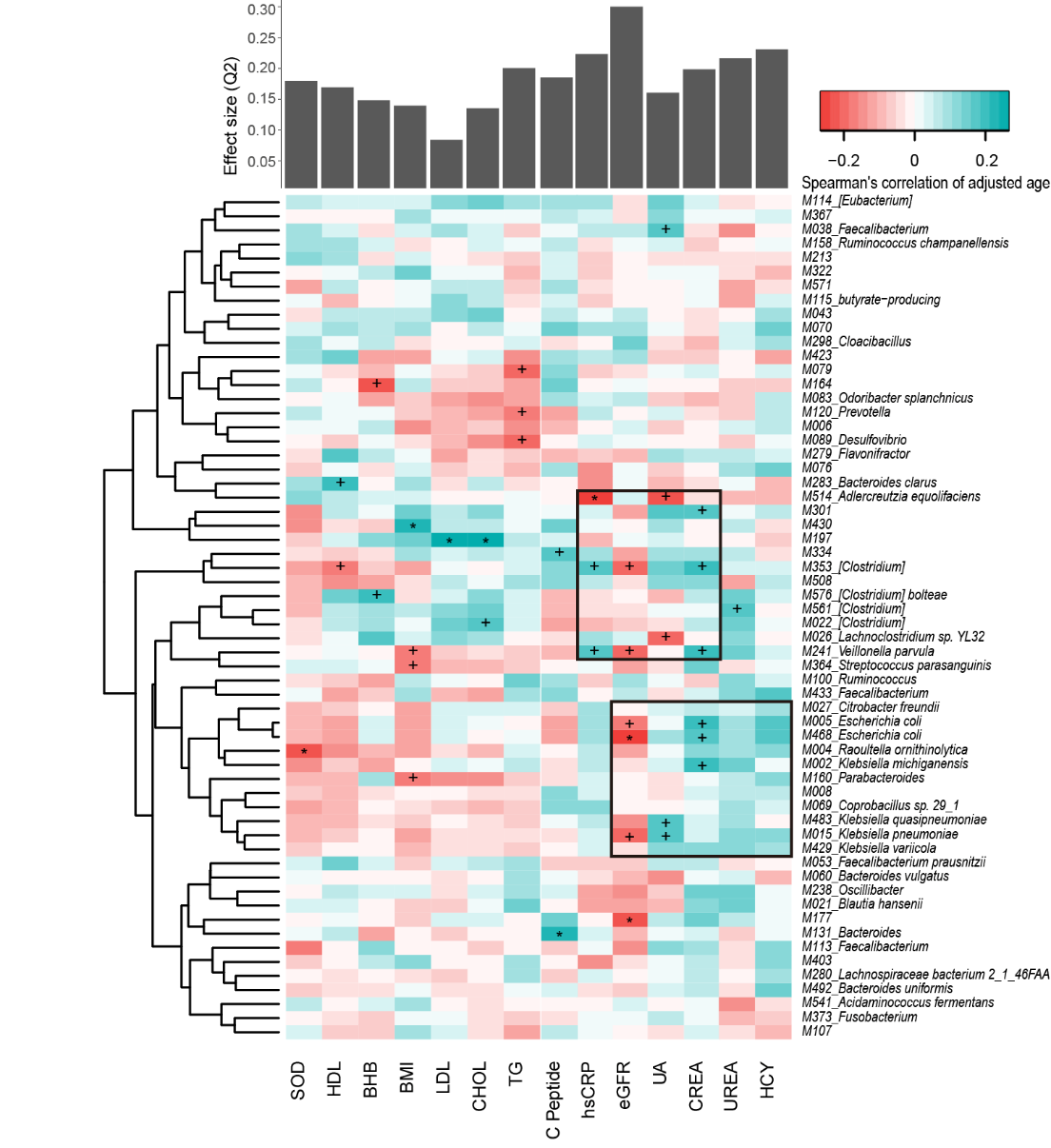


**Figure S11. Relative abundances of the MGSs associated with the uremic toxins or SBAs in the predictive models.** MGSs are highly correlated with kidney-associated clinical parameters. Spearman correlation coefficients between the MGSs and the kidney-associated clinical parameters are displayed as heat maps. ‘+’, *P*< 0.05; ‘*’, *P* < 0.01; ‘**’, *P* < 0.001. The overall effect size of microbes on each clinical parameter is shown at the top.


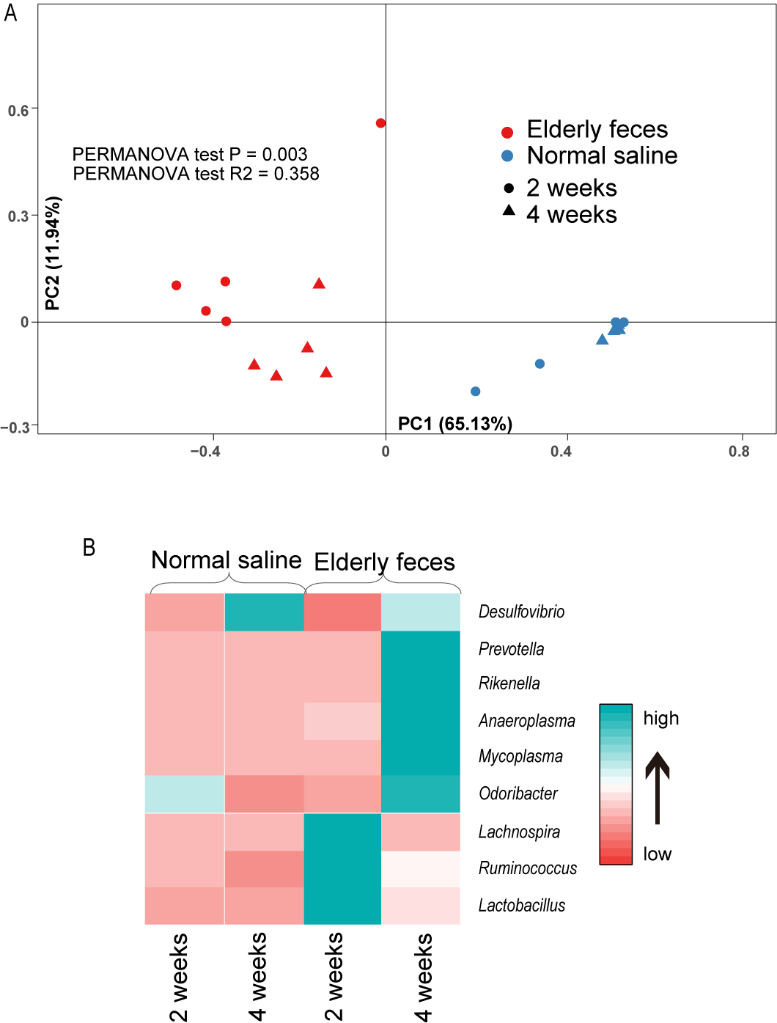


**Figure S12. Differences in gut microbiota between mice gavaged with saline and mice gavaged with elderly human feces in FMT experiments.** (A) PCA analysis shows a clear separation between the gut microbiome of mice gavaged with saline and those gavaged with elderly human feces. (B) Differences in gut microbiota between mice gavaged with saline and those gavaged with elderly human feces, as well as changes in their metabolite levels at different time points (p<0.1).

**
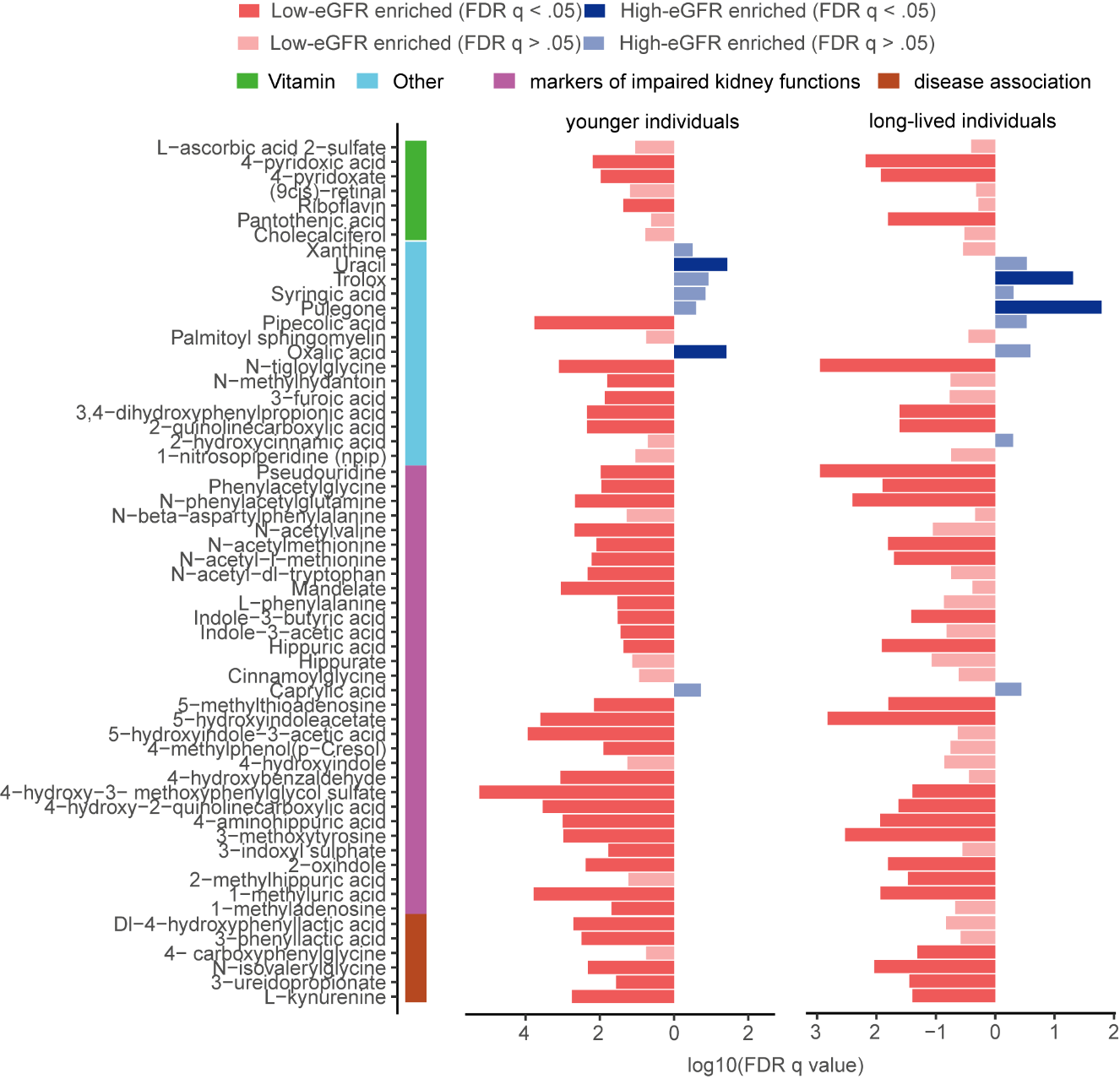
**

**Figure S13. Serum metabolites related to eGFR in younger and long-living individuals.**

**Captions for Supplementary Table S1-16**

**(Tables S1-16 as separate file)**

**Table S1.** Summary of host data from the Guangxi longevity, Yunnan aging and Japan aging cohorts.

**Table S2.** Summary of telomere length from the Guangxi longevity cohort.

**Table S3.** Detailed information on the gut microbiome sequencing data, genome assembly, gene predictions, and percentage of reads mapped to the non-redundant gene catalog (4.4 M).

**Table S4.** Summary information of 601 MGSs generated in this study.

**Table S5.** The main driving bacteria of the different enterotypes.

**Table S6.** Age-related metagenomic species (MGS and metaphlan).

**Table S7.** Age-related metabolites in the Guangxi longevity and Yunnan aging cohorts.

**Table S8.** Detailed information on the effect size of the gut microbiome and host phenome variables on the variation in serum metabolomes is shown in Supplementary Figure 6.

**Table S9.** Key enzymes involved in kidney toxin metabolism and bile acid metabolism used in this study.

**Table S10.** Changes in key enzymes involved in renal toxin metabolism and bile acid metabolism with age at different MGSs in this study.

**Table S11.** Detailed information on the random forest models used to predict the concentration of serum metabolites.

**Table S12.** Detailed information on the random forest models used to predict the concentrations of serum metabolites.

**Table S13.** Detailed information of the random forest models used to predict the host phenomic indices.

**Table S14.** Mediation linkages among microbiome, metabolites and age.

**Table S15.** Power calculations of the correlation analysis.

**Table S16.** Differences in age-related serum metabolites between the high-eGFR and low-eGFR groups in younger and long-living individuals.
